# Species Diversity, Mating Strategy and Pathogenicity of *Calonectria* Species from Diseased Leaves and Soils in the *Eucalyptus* Plantation in Southern China

**DOI:** 10.3390/jof7020073

**Published:** 2021-01-20

**Authors:** WenXia Wu, ShuaiFei Chen

**Affiliations:** 1 China Eucalypt Research Centre (CERC), Chinese Academy of Forestry (CAF), Zhanjiang 524022, China; wuwenxia_hainan@126.com; 2 Nanjing Forestry University (NJFU), Nanjing 210037, China

**Keywords:** *Cylindrocladium*, forest pathogens, fungal ecology, leaf blight, multi-gene phylogeny, tree disease

## Abstract

Many *Calonectria* species are causal agents of diseases on several forestry, agricultural and horticultural crops. *Calonectria* leaf blight is one of the most important diseases associated with *Eucalyptus* plantations and nurseries in Asia and South America. Recently, symptoms of leaf rot and leaf blight caused by *Calonectria* species were observed in a one-year-old *Eucalyptus* experimental plantation in GuangXi Province, southern China. To better understand the species diversity, mating strategy and pathogenicity of *Calonectria* species isolated from diseased tissues and soils, diseased leaves and soils under the trees from ten *Eucalyptus urophylla* hybrid genotypes were collected. Three hundred and sixty-eight *Calonectria* isolates were obtained from diseased *Eucalyptus* leaves and soils under these trees, and 245 representative isolates were selected based on the sampling substrates and *Eucalyptus* genotypes and identified by DNA sequence analyses based on the translation elongation factor 1-alpha (*tef1*), β-tubulin (*tub2*), calmodulin (*cmdA*) and histone H3 (*his3*) gene regions, as well as a combination of morphological characteristics. These isolates were identified as *Calonectria hongkongensis* (50.2%), *C. pseudoreteaudii* (47.4%), *C. aconidialis* (1.6%), *C. reteaudii* (0.4%) and *C. auriculiformis* (0.4%). This is the first report of *C. reteaudii* and *C. auriculiformis* occurrence in China. *Calonectria pseudoreteaudii* was isolated from both *Eucalyptus* diseased leaves and soils; the other four species were only obtained from soils. *MAT1-1-1* and *MAT1-2-1* gene amplification and mating type assignment results showed that *C. pseudoreteaudii* is heterothallic and an asexual cycle represents the primary reproductive mode, *C. reteaudii* and *C. auriculiformis* are likely to be heterothallic and *C. hongkongensis* and *C. aconidialis* are homothallic. Based on the genetic diversity comparisons for *C. pseudoreteaudii* isolates from diseased leaves and soils, we hypothesize that *C. pseudoreteaudii* in soils was spread from diseased leaves. Both the mycelia plug and conidia suspension inoculations indicated that all five *Calonectria* species were pathogenic to the two *Eucalyptus* genotypes tested and the tolerance of the two genotypes differed. It is necessary to understand the ecological niche and epidemiological characteristics of these *Calonectria* species and to select disease resistant *Eucalyptus* genotypes in southern China in the future.

## 1. Introduction

Currently, the *Eucalyptus* (Myrtaceae, Myrtales) plantation area has expanded to more than 5.4 million hm^2^ in China, accounting for nearly 2.5% of the national total forestry area. China’s *Eucalyptus* plantations produce more than 30 million m^3^ timber per year, accounting for more than 33% of China’s total domestic timber production [1]. Therefore, *Eucalyptus* plantations play an important role in wood supply in China. *Eucalyptus* plantations are mainly distributed in GuangXi, GuangDong, YunNan, FuJian, SiChuan and HaiNan Provinces in southern China. Of these, GuangXi Province has the largest area of *Eucalyptus* [1,2].

With the extensive development of *Eucalyptus* plantations over the past 30 years, pathogens and pests have rapidly emerged as a significant threat to *Eucalyptus* plantations in China [2,3]. Important diseases in *Eucalyptus* plantations include bacterial wilt caused by *Ralstonia pseudosolanacearum* [4,5]; stem canker/wilt caused by species of Botryosphaeriaceae [6,7,8], *Cryphonectriaceae* [9,10,11], *Ceratocystis* [12,13] and *Teratosphaeria zuluensis* [14,15]; and leaf spot/blight caused by Mycosphaerellaceae and Teratosphaeriaceae species [16,17], *Calonectria* [18,19,20] and *Quambalaria* [21,22]. Of these, leaf blight caused by *Calonectria* species is considered to be one of the most important diseases in *Eucalyptus* plantations in southern China [3,18,20].

The genus *Calonectria* includes important plant pathogens infecting more than 335 plant species, distributed by nearly 100 plant families. These plants include forestry, agricultural and horticultural crops [23,24,25]. In forestry, *Calonectria* species mainly attack the families Fabaceae (*Acacia* spp.), Myrtaceae (*Eucalyptus* spp.) and Pinaceae (*Pinus* spp.) [23,24]. In *Eucalyptus*, this fungus causes stem and leaf rot in nurseries, leaf and shoot blight and stem canker in plantation [5,20,23]. These diseases are mainly reported in Asia, Africa and South America [20,26,27].

To date, 23 species of *Calonectria* have been identified and described based on DNA sequence data in China [20,28,29]. Of these, 15 species, *C. aciculata*, *C. crousiana*, *C. eucalypti*, *C. fujianensis*, *C. hawksworthii*, *C. pauciramosa*, *C. cerciana*, *C. pseudoreteaudii*, *C. aconidialis*, *C. asiatica*, *C. honghensis*, *C. hongkongensis*, *C. kyotensis*, *C. lateralis* and *C. yunnanensis* have been isolated from *Eucalyptus* plants or the soils in southern China. The first eight of these species were isolated from infected tissues (leaves, shoots or branches) in plantations, while the latter nine species were from soils (*C. cerciana* and *C. pseudoreteaudii* were isolated from both *Eucalyptus* tissues and soils) [20,28,29]. Additionally, *C. cerciana*, *C. pauciramosa* and *C. pseudoreteaudii* were also isolated from diseased seedlings in nurseries [19,29,30] and *C. pseudoreteaudii* from soils in a *Eucalyptus* nursery [19]. Our previous research results of conidia suspension inoculations in *Eucalyptus* seedlings showed that *C. crousiana*, *C. eucalypti*, *C. fujianensis*, *C. pauciramosa* and *C. pseudoreteaudii* are all pathogenic to all the tested *Eucalyptus* genotypes [18,20]. All the five tested species were originally isolated from diseased *Eucalyptus* tissues in plantations or nurseries [18,20].

Previous research results indicated that a relatively large number of *Calonectria* species are distributed in *Eucalyptus* plantations, both in diseased tissues and soils under these trees. However, the differences of species diversity, mating strategy and pathogenicity of these fungi isolated from diseased tissues and soils remain unknown. Recently, leaf blight caused by *Calonectria* in one *Eucalyptus* experimental plantation in GuangXi Province was observed, the diseased leaves from *Eucalyptus* trees and soils under these trees were collected, and the *Calonectria* fungi were isolated. The aims of this study were to (i) identify the *Calonectria* fungi based on multi-gene phylogeny and morphological characteristics, (ii) compare the species diversity between isolates obtained from diseased leaves and soils, (iii) test the mating type of obtained *Calonectria* species, and (iv) test the pathogenicity of *Calonectria* species obtained from both diseased leaves and soils.

## 2. Materials and Methods

### 2.1. Disease Survey Site, Sample Collection and Fungal Isolation

**Figure 1:**
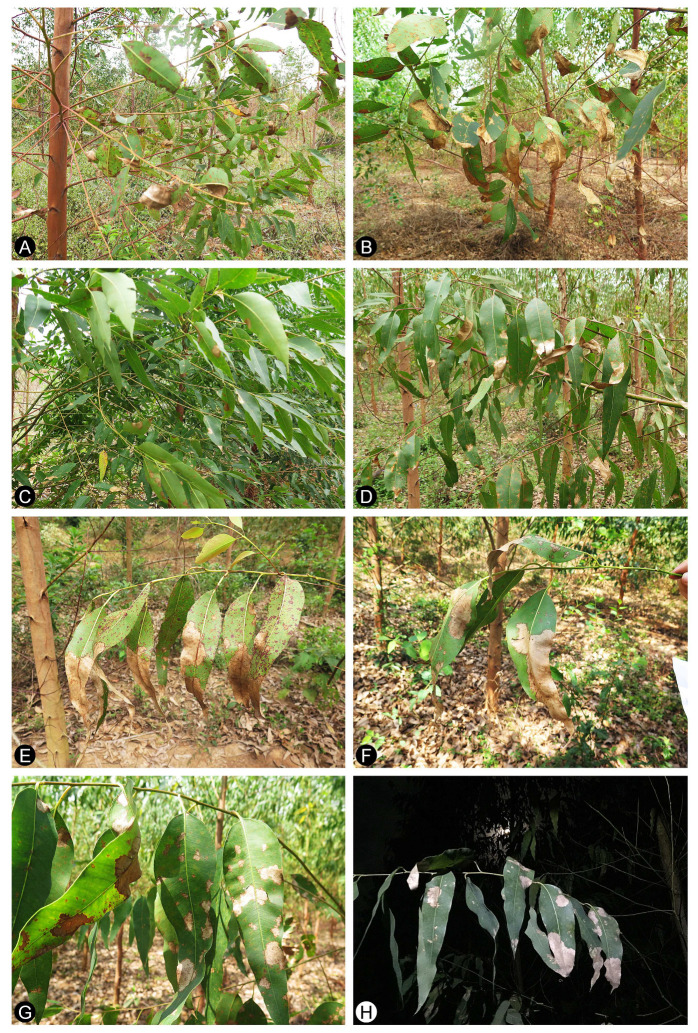
Disease symptoms on multiple *Eucalyptus* genotypes in one experimental plantation caused by species of *Calonectria*. (**A**–**C**): Leaf spot in three *E. urophylla* × *E. grandis* hybrid genotypes CEPT1863 (**A**), CEPT1861 (**B**), and CEPT1862 (**C**), the infected leaves of CEPT1861 and CEPT1862 became blighted and dried; (**D**–**H**): Leaf spot and blight in three *E. urophylla* × *E. tereticornis* hybrid genotypes, CEPT1866 (**D**,**E**), CEPT1868 (**F**), and CEPT1869 (**G**,**H**).

The disease survey was conducted in a one-year-old *Eucalyptus* breeding experimental plantation in the BeiHai region, GuangXi Province, southern China (21°33′19.8756″ N, 109°42′27.0792″ E) in October, 2018. Ten *Eucalyptus* genotypes were planted in the experimental plantation. These included six *Eucalyptus urophylla* × *E. grandis* hybrid genotypes (CEPT1860–CEPT1865) and four *E. urophylla* × *E. tereticornis* hybrid genotypes (CEPT1866–CEPT1869). All ten *Eucalyptus* genotypes were naturally infected by *Calonectria* species (Figure 1).

Diseased leaves with typical symptoms caused by *Calonectria* species were collected from 13 to 20 trees for each of the ten *Eucalyptus* hybrid genotypes, depending on the planted areas of each genotype. Soil samples under each sampled diseased tree were also collected. These samples of diseased leaves and soils were transported to the laboratory for isolation, morphological examination, and further molecular research.

To induce *Calonectria* sporulation, diseased leaves were placed in moist dishes (diameter 70 mm, height 16 mm; tissue paper moistened with sterile water) at room temperature and incubated for 1–3 days. Soil samples were baited with *Medicago sativa* (alfalfa) germinating seeds using the method described by Crous [23]. Fungal isolates with typical morphological characteristics of *Calonectria* were isolated from diseased leaves and soil samples. The conidia masses were transferred to 2% (*v*/*v*) malt extract agar (MEA) (20 g malt extract powder and 20 g agar powder per liter of water: malt extract powder was obtained from the Beijing Shuangxuan microbial culture medium products factory, Beijing, China; the agar powder was obtained from Beijing Solarbio Science & Technology Co., Ltd., Beijing, China) with a sterile needles under stereoscopic microscope and incubated for 3–5 days. To obtain pure cultures, a single hyphal tip from each culture was transferred to 2% MEA plates and incubated at room temperature for 7–10 days. The pure cultures were deposited in the culture collection (CSF) at the China Eucalypt Research Centre (CERC) of the Chinese Academy of Forestry (CAF) in ZhanJiang, GuangDong Province, China.

### 2.2. DNA Extraction, PCR Amplification and Sequencing

Representative isolates were selected based on the sampling substrates and *Eucalyptus* genotypes for DNA extraction and sequence comparisons. DNA was extracted from 10-day-old cultures and mycelia were collected using a sterilized scalpel and transferred to 2 mL Eppendorf tubes. Total genomic DNA was extracted following the CTAB protocol described by van Burik and co-authors [31]. The extracted DNA was dissolved using 30 µL TE buffer (1 M Tris-HCl and 0.5 M EDTA, pH 8.0), and 3 µL RNase (10 mg/mL) was added at 37 °C for 1 h to degrade RNA. Finally, DNA concentration was measured with a Nano-Drop 2000 spectrometer (Thermo Fisher Scientific, Waltham, MA, USA).

According to previous research results, sequences of partial gene regions of translation elongation factor 1-alpha (*tef1*), β-tubulin (*tub2*), calmodulin (*cmdA*), and histone H3 (*his3*) were used to successfully identify *Calonectria* species [26,28,32]. These four partial gene regions were amplified using the primer pairs EF1-728F/EF2, T1/CYLTUB1R, CAL-228F/CAL-2Rd and CYLH3F/CYLH3R, respectively, the PCR procedure was conducted as described by Liu and Chen [33], Lombard and co-authors [30] (Table 1).

To obtain accurate sequences for each sequenced isolates, all PCR products were sequenced in forward and reverse directions by the same primers used for PCR amplification by the Beijing Genomics Institute, Guangzhou, China. All sequences obtained in this study were edited using MEGA v. 6.0.5 software [34] and were deposited in GenBank (https://www.ncbi.nlm.nih.gov).

**Table 1:** Primers for amplification of *tef1*, *tub2*, *cmdA*, *his3* and mating type gene fragments.

Target Gene	Primer Name	Primer Sequence (5′ to 3′)	Tm (°C)	Fragment Size (bp)	Reference
translation elongation factor 1-alpha (*tef1*)	EF1-728F	CATCGAGAAGTTCGAGAAGG	52	500	[30,33]
	EF2	GGA(G/A)GTACCAGT(G/C)ATCATGTT			[30,33]
β-tubulin (*tub2*)	T1	AACATGCGTGAGATTGTAAGT	52	520	[30,33]
	CYLTUB1R	AGTTGTCGGGACGGAAGAG			[30,33]
calmodulin (*cmdA*)	CAL-228F	GAGTTCAAGGAGGCCTTCTCCC	55	470	[30,33]
	CAL-2Rd	TGRTCNGCCTCDCGGATCATCTC			[30,33]
histone H3 (*his3*)	CYLH3F	AGGTCCACTGGTGGCAAG	55	450	[30,33]
	CYLH3R	AGCTGGATGTCCTTGGACTG			[30,33]
*MAT1-1-1*	Cal_MAT111_F	ATGCTTCCTCAGTCTTTGCT	53	330	[35]
	Cal_MAT111_R	CTTGAAYRGGGTTGGTGG			[35]
*MAT1-2-1*	Cal_MAT121_F	GCAAGGAYCGCCACCRAAT	58	240	[35]
	Cal_MAT121_R	GACACCTCKGCGTTTCTTCTCAG			[35]

### 2.3. Multi-Gene Phylogenetic Analyses

To preliminarily identify the species which the isolates obtained in this study, a standard nucleotide BLAST search was conducted using the *tef1*, *tub2*, *cmdA* and *his3* sequences. The sequences of *tef1*, *tub2*, *cmdA* and *his3* gene regions generated in this study were compared with sequences of type specimen strains of published *Calonectria* species for phylogenetic analyses. Sequences of all the published species in the relative species complexes were used for sequence comparisons and phylogenetic analyses. The datasets of Liu and co-authors [28] were used as templates for analyses.

Sequences of each of the *tef1*, *tub2*, *cmdA* and *his3* gene regions as well as the combination of these four gene regions were aligned using the online version of MAFFT v. 7 (http://mafft.cbrc.jp/alignment/server) with the alignment strategy FFT-NS-i (Slow; interactive refinement method). After initial alignments, sequence alignments were manually edited using MEGA v. 6.0.5 software [34].

Maximum parsimony (MP) and maximum likelihood (ML) were used frequently for phylogenetic analyses of *Calonectria* species [28,29,36]. To test whether the analysis results between the two methods are consistent, both MP and ML were used for phylogenetic analyses for sequence datasets of each of the four genes and the combination of four gene regions. The MP and ML analyses were conducted using the methods described by Liu and Chen [33]. Phylogenetic trees were viewed using MEGA v. 6.0.5 [34]. Sequence data of two isolates of *Curvicladiella cignea* (CBS 109167 and CBS 109168) were used as outgroups [28].

### 2.4. Morphology

The representative isolates of each *Calonectria* species identified by DNA sequence comparisons were selected for morphological description. The size of macroconidia and width of vesicles are the most typical asexual characteristics used for morphological comparison in *Calonectria* [19,29,33]. The asexual structures of selected *Calonectria* isolates were induced in synthetic nutrient-poor agar (SNA) [37] following the method described by Liu and Chen [33]. Fifty measurements of macroconidia and vesicles were made for the selected isolates.

### 2.5. MAT Gene Amplification and Mating Type Assignment

To further understand the possible reproductive mode of the population of each *Calonectria* species identified in this study, the mating type idiomorph of each representative *Calonectria* isolate of an identified species was identified. Mating type primer pairs Cal_MAT111_F/Cal_MAT111_R and Cal_MAT121_F/ Cal_MAT121_R were used to amplify the *MAT1-1-1* and *MAT1-2-1* genes in all selected isolates using the protocol described by Li and co-authors [35] (Table 1). For the heterothallic *Calonectria* species based on *MAT* gene amplification results, the species with an adequate number of isolates as one population, the possibility that recombination had taken place was tested. A two-tailed exact binomial method [38] was used, and each population that included isolates of both mating types was tested in R version 3.6.1 to evaluate whether the MAT1-1 and MAT1-2 frequency significantly deviated from a 1:1 ratio.

### 2.6. Pathogenicity Tests

To determine the pathogenicity of *Calonectria* species obtained in this study, representative isolates of all *Calonectria* species isolated from diseased leaves and soils were identified by phylogenetic analyses, and morphological characteristics were selected for inoculation trials. Two *Eucalyptus* genotypes, *E. urophylla* × *E. tereticornis* hybrid genotype CEPT1876 and *E. urophylla* × *E. grandis* hybrid genotype CEPT1877 were selected for inoculations. The inoculated *Eucalyptus* seedlings were three months old and approximately 40 cm tall.

In this study, the inoculations were conducted with both mycelia plug and conidia suspension of selected *Calonectria* isolates. All the inoculated seedlings were in similar size. In the mycelia plug inoculations, for each *Eucalyptus* genotype, mycelia plugs of each isolate were inoculated on ten leaves of two to three *Eucalyptus* seedlings, and ten leaves of other two to three *Eucalyptus* seedlings treated with sterile MEA plugs were regarded as negative controls. For inoculation, mycelia plugs (5 mm diameter) from 7-day-old MEA cultures were placed upside down on the abaxial surface of the leaflets. In the conidia suspension inoculations, the conidia suspensions for each isolate were prepared using the method described in Graça and co-authors [39] and Wang and Chen [20]. The conidia suspensions prepared for each isolates were measured using a hemocytometer, being the concentration adjusted to 5 × 10^4^ conidia/mL. For each isolate, eight seedlings of each genotype were inoculated by spraying the conidia suspension until the suspension run off the leaves. Sterile water was sprayed onto other eight seedlings as the negative control using the same treatment. To allow sufficient humidity for infection development, the *Eucalyptus* seedlings inoculated with mycelia plug and conidia suspension, were maintained in plastic chambers (length: 190 cm, width: 90 cm, height: 63 cm) with intermittent water nebulization for 30 s at three-hour intervals and were maintain stable climatic conditions (temperature 24–26 °C; humidity 60–70%) for three days. The experiments using both mycelia plug and conidia suspension were all repeated once using the same methodology.

The plastic chambers were removed three days after inoculation. For mycelia plug inoculations, the length of lesions produced was measured. For conidia suspension inoculations, the disease index (DI) was calculated. Leaf disease severity was assessed by estimating the percentage of lesioned area on each leaf with a scale from 0 to 5, where 0 indicated no lesions, 1 indicated that 1 to 10% area of the leaf was lesioned, 2 indicated that 11 to 25% area of the leaf was lesioned, 3 indicated that 26 to 50% area of the leaf was lesioned, 4 indicated that 51 to 75% area of the leaf was lesioned, and 5 indicated that 76 to 100% area of the leaf was lesioned. The DI was calculated according to Mishra and co-authors [40]. The percentage of lesioned area caused by *Calonectria* isolate on each leaf of inoculated *Eucalyptus* seedlings was calculated through the software “Leaf Doctor” [41].

For re-isolations, small pieces of discolored leaf (approximately 0.04 cm^2^) from the edges of the resultant lesions were cut and placed on 2% MEA at room temperature. Re-isolations were conducted for randomly selected leaves from four randomly selected seedlings of each *Eucalyptus* genotype for each inoculated isolate, and the randomly selected leaves from all seedlings were inoculated as negative controls. Re-isolations were conducted for both mycelia plug and conidia suspension inoculations. The re-isolated fungi were identified and confirmed by morphological characteristics of culture, macroconidiophore and macroconidia, as well as the disease symptoms produced on the leaves with the original fungi used for inoculations. Statistical analyses were performed using SPSS Statistics 22 software (IBM Corp., Armonk, NY, USA) by one-way analysis of variance (ANOVA) for mycelia plug and conidia inoculation results, respectively. The inoculations were conducted in September, 2020 at the experimental nursery of China Eucalypt Research Centre, GuangDong Province, China.

## 3. Results

### 3.1. Disease Symptoms, Sample Collection and Fungal Isolation

Disease symptoms observed in the present study include greyish water-soaked spots on the leaves of the lower branches on the infected trees in the early stage (Figure 1). These spots subsequently form extensive necrotic areas, and leaves become dry and curly (Figure 1A,B,E,F). White masses of conidiophores with typical morphological characteristics of *Calonectria* species are frequently observed on the shoots and leaves of *Eucalyptus* trees. Different *Eucalyptus* genotypes infected by pathogens show different degrees of susceptibility and symptoms (Figure 1). The disease symptoms observed in this study were similar to those caused by *Calonectria* species in *Eucalyptus* trees as reported previously in China [20]. These samples of diseased leaves and soils were transported to the laboratory for isolation, morphological examination and further molecular research. Diseased leaf samples were collected from 13 to 20 trees of each of ten *Eucalyptus* genotypes. A total of 190 diseased leaf samples with white masses of conidiophores with typical morphological characteristics of *Calonectria* species were obtained from 190 diseased trees; furthermore, 190 soil samples were collected from soils under these trees (Table 2). For diseased leaf samples, *Calonectria* was successfully isolated from all sampled diseased trees of each of the ten *Eucalyptus* genotypes, with the exception of *Eucalyptus* genotypes CEPT1862, CEPT1863, CEPT1865 and CEPT1866. Finally, *Calonectria* was isolated from 184 of the 190 diseased trees (Table 2). One to two *Calonectria* isolates, depending on the variation of conidia morphology, from each sampled tree were isolated, and a total of 186 *Calonectria* isolates were obtained from sampled trees (Table 2). For soil samples, no *Calonectria* was isolated from soils sampled under *Eucalyptus* genotype CEPT1863; *Calonectria* isolates were obtained from two to 12 soil samples collected from the soil under each of the other nine *Eucalyptus* genotypes. In total, *Calonectria* was isolated from 47 of the 190 soil samples (Table 2). One to six *Calonectria* isolates were isolated from each of the soil samples in which *Calonectria* was induced and sporulated. In all, 182 *Calonectria* isolates were obtained from the 47 soil samples (Table 2). Three hundred and sixty-eight *Calonectria* isolates were obtained from diseased *Eucalyptus* trees and soils under these trees (Table 2).

### 3.2. Sequencing

Sixty-three *Calonectria* isolates obtained from diseased trees of ten *Eucalyptus* genotypes (four to 12 isolates from each *Eucalyptus* genotype), and all 182 isolates obtained from soils were used for DNA extraction and sequence comparisons (Appendix A Table A1). The *tef1* and *tub2* genes were amplified for all 245 isolates. Subsequently, 73 representative isolates were selected based on *tef1* and *tub2* sequences so as to include all the genotypes revealed by these two loci, as well as all the sampled *Eucalyptus* genotypes and substrate. The *cmdA* and *his3* loci were then also sequenced for these 73 isolates (Appendix A Table A1).

### 3.3. Multi-Gene Phylogenetic Analyses

The sequence fragments were approximately 500 bp for *tef1*, 565 bp for *tub2*, 685 bp for *cmdA* and 440 bp for *his3*. Based on the sequences of *tef1*, *tub2*, *cmdA* and *his3* loci, the 73 representative isolates represented 11 genotypes. Forty isolates representing all 11 genotypes that were isolated from the diseased leaves and soils associated with all the relative *Eucalyptus* genotypes were selected for phylogenetic analyses (Appendix A Table A1). Results of the standard nucleotide BLAST search conducted using the *tef1*, *tub2*, *cmdA* and *his3* sequences showed that the isolates obtained in the current study belong three species complexes of *Calonectria*, including *C. reteaudii* species complex, the *C. cylindrospora* species complex, and the *C. kyotensis* species complex. Based on the recently published results in Liu and co-authors [28], sequences of *tef1*, *tub2*, *cmdA* and *his3* published species in the *C. reteaudii* species complex, *C. cylindrospora* species complex and *C. kyotensis* species complex, respectively, were used for sequence comparisons and phylogenetic analyses (Appendix A Table A2).

**Figure 2:**
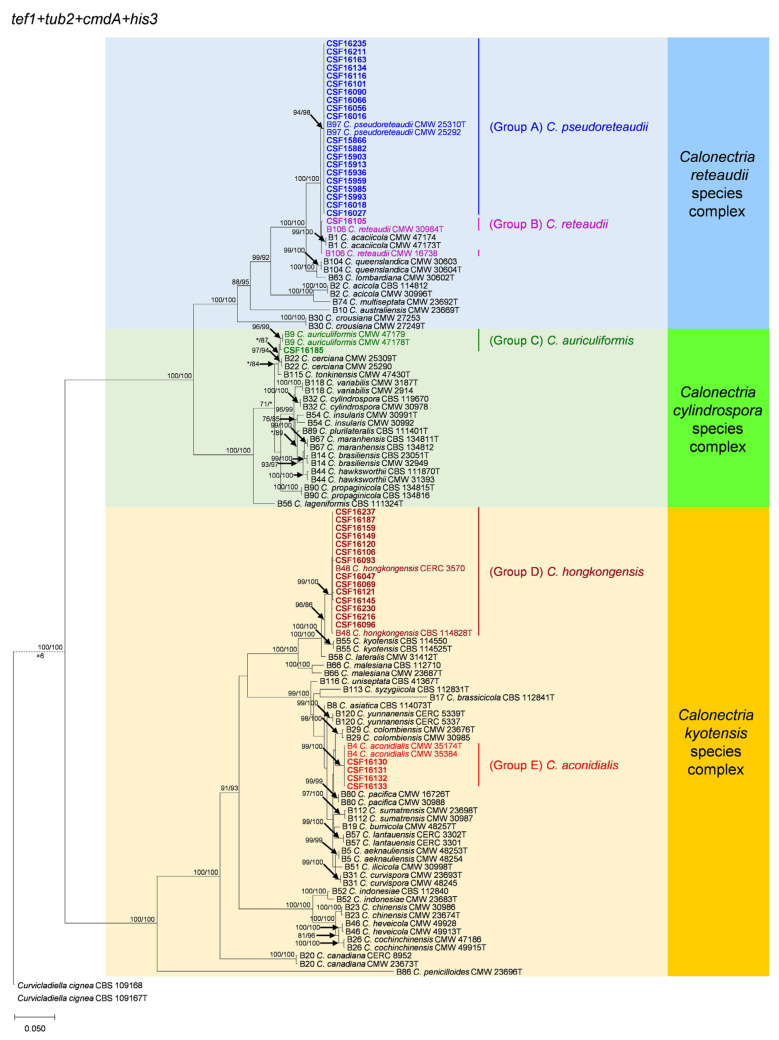
Phylogenetic tree of *Calonectria* species based on maximum likelihood (ML) analyses of the dataset of combined *tef1*, *tub2*, *cmdA* and *his3* gene sequences in this study. Bootstrap support values ≥70% are presented above the branches as follows: ML/MP. Bootstrap values <70% and absent are marked with “*”. Isolates highlighted in five different colors and bold were obtained in this study. Ex-type isolates are marked with “T”. The “B” species codes are consistent with the recently published results in Liu and co-authors [28]. The *Curvicladiella cignea* (CBS 109167 and CBS 109168) was used as outgroup taxon.

The partition homogeneity test (PHT) comparing the combination of *tef1*, *tub2*, *cmdA* and *his3* gene datasets generated a *p*-value of 0.001, indicating the accuracy of the combined datasets did not suffer relative to the individual partitions [42], sequences of the four loci were combined for analyses. For the phylogenetic trees based on *tef1*, *tub2*, *cmdA* and *his3* individually and the combined sequence datasets, the overall topologies were similar, but the relative position of some *Calonectria* species differed slightly between the MP and ML trees. The five ML trees are presented in Figure 2, Supplementary Figures S1–S4. The numbers of taxa and parsimony informative characters, statistical values for the MP analyses, and parameters for the best-fit substitution models of ML analyses are provided in Table 3.

Sequence data were not available for *tub2* for four isolates (CSF16130, CSF16131, CSF16132 and CSF16133) obtained in this study and ex-type isolates of various published *Calonectria* species (Appendix A Table A1 and Table A2). The 40 *Calonectria* isolates were clustered in five groups (Group A, Group B, Group C, Group D and Group E) based on *tef1*, *his3* and combined *tef1*/*tub2*/*cmdA*/*his3* analyses (Figure 2; Supplementary Figures S1 and S4). These isolates clustered in four groups based on *tub2* analyses, including Groups A, B, C, and D, where sequences of Group E were not available (Supplementary Figure S2); and four groups were based on *cmdA* analyses, including Groups C, D and E, where Group A clustered with Group B (Supplementary Figure S3).

The phylogenetic analyses showed that isolates in Group A and Group B belong the *C. reteaudii* species complex. Isolates in Group A clustered with *C. pseudoreteaudii* based on phylogenetic analyses of *tef1*, *tub2* and *his3* datasets (Supplementary Figures S1, S2 and S4) and clustered with *C. pseudoreteaudii* and *C. reteaudii* in the *cmdA* tree (Supplementary Figure S3). In the combined *tef1*/*tub2*/*cmdA*/*his3* tree, these isolates were clustered with *C. pseudoreteaudii* (Figure 2). Isolates in Group A were identified as *C. pseudoreteaudii*. Isolate CSF16105 in Group B clustered with *C. reteaudii* in the *tub2* and *his3* trees (Supplementary Figures S2 and S4), with *C. reteaudii* and *C. acaciicola* in the *tef1* tree (Supplementary Figure S1), and with *C. reteaudii* and *C. pseudoreteaudii* in the *cmdA* tree (Supplementary Figure S3). The isolate was clustered with *C. reteaudii* in the combined *tef1*/*tub2*/*cmdA*/*his3* tree (Figure 2). The isolate in Group B was identified as *C. reteaudii*.

Isolate CSF16185 in Group C belongs the *C. cylindrospora* species complex. This isolate clustered with *C. auriculiformis* in the *tef1* tree (Supplementary Figure S1); clustered with *C. cerciana* and was closely related to *C. tonkinensis*, *C. lageniformis* and *C. auriculiformis* in the *tub2* trees (Supplementary Figure S2); clustered with *C. lageniformis* and was closely related to *C. cerciana*, *C. tonkinensis* and *C. auriculiformis* in the *cmdA* tree (Supplementary Figure S3) and clustered with *C. auriculiformis*, *C. cerciana* and *C. tonkinensis* in the *his3* tree (Supplementary Figure S4). This isolate was most closely related to *C. auriculiformis* in the combined *tef1*/*tub2*/*cmdA*/*his3* tree (Figure 2). The Isolate in Group C was identified as *C. auriculiformis*.

All the isolates in Group D and Group E belong the *C. kyotensis* species complex. Isolates in Group D were clustered with *C. hongkongensis* in each of the *tef1*, *tub2*, *cmdA*, *his3* and the combined *tef1*/*tub2*/*cmdA*/*his3* trees (Figure 2, Supplementary Figures S1–S4). These isolates were identified as *C. hongkongensis*. The *tub2* sequences are not available for the four isolates in Group E; these isolates were clustered with *C. aconidialis* in each of the *tef1*, *cmdA*, *his3* and the combined *tef1*/*tub2*/*cmdA*/*his3* trees (Figure 2, Supplementary Figures S1, S3 and S4). Isolates in Group E were identified as *C. aconidialis*.

### 3.4. Species and Genetic Diversity Associate with Eucalyptus Genotype and Substrate

Based on the sequence comparisons of *tef1*, *tub2*, *cmdA* and *his3* sequences, the *Calonectria* isolates sequenced were identified as *C. hongkongensis* (123), *C. pseudoreteaudii* (116), *C. aconidialis* (4), *C. reteaudii* (1) and *C. auriculiformis* (1). With the exception of *C. pseudoreteaudii*, which was isolated from both *Eucalyptus* diseased leaves and soils, the other four species were only obtained from soils under the *Eucalyptus* trees (Appendix A Table A1). Sixty-three isolates of *C. pseudoreteaudii* were isolated from diseased leaves from all the ten sampled *Eucalyptus* genotypes; 53 isolates of *C. pseudoreteaudii* and all the 123 isolates of *C. hongkongensis* were isolated from soils under the same nine of the ten *Eucalyptus* genotypes (except for CEPT1863) (Appendix A Table A1). *Calonectria reteaudii*, *C. auriculiformis* and *C. aconidialis* were only isolated from *Eucalyptus* genotypes CEPT1864, CEPT1868 and CEPT1865, respectively (Appendix A Table A1).

The genotypes of *Calonectria* species identified were determined by sequences of *tef1*, *tub2*, *cmdA* and *his3*; the results indicated they were generated from *C. pseudoreteaudii* (2), *C. reteaudii* (1), *C. auriculiformis* (1), *C. hongkongensis* (6) and *C. aconidialis* (1) (Appendix A Table A1, Table 4). For *C. pseudoreteaudii*, 115 of all 116 isolates presented the same genotype (Genotype 1 of *C. pseudoreteaudii*) (Appendix A Table A1), which was distributed in diseased leaves of all ten *Eucalyptus* genotypes and soils under nine *Eucalyptus* genotypes; only one isolate presented a different genotype (CSF16016, Genotype 2 of *C. pseudoreteaudii*), which was isolated from diseased leaves of CEPT1868 (Appendix A Table A1, Table 4). Ninety-eight of the 123 isolates of *C. hongkongensis* presented the same genotype (*C. hongkongensis* Genotype 1). This genotype was dominant in *C. hongkongensis,* and these 98 isolates were isolated from soils under eight of the ten *Eucalyptus* genotypes (Appendix A Table A1, Table 4).

### 3.5. Morphology

Based on phylogenetic analysis results, 16 isolates represented five *Calonectria* species (*C. pseudoreteaudii*: CSF15985, CSF16016, CSF16018, CSF16027, CSF16056, CSF16102 and CSF16116; *C. reteaudii*: CSF16105; *C. auriculiformis*: CSF16185; *C. hongkongensis*: CSF16121, CSF16145, CSF16230 and CSF16237; *C. aconidialis*: CSF16130, CSF16131 and CSF16133) identified in this study were selected for macroconidia and vesicle morphological comparisons (Appendix A Table A1, Table 5). These isolates can be distinguished into three groups based on the vesicle shape. Isolates of *C. pseudoreteaudii* and *C. reteaudii* produce clavate or narrowly clavate vesicles; the vesicles of *C. auriculiformis* are ellipsoidal to fusiform to obpyriform, and the vesicles of *C. hongkongensis* and *C. aconidialis* are pyriform to sphaeropedunculate, ovoid to sphaeropedunculate and sphaeropedunculate. With the exception of *C. reteaudii* and *C. auriculiformis*, in which only one isolate was studied for each of the two species, the average of measurements showed that significant variations exist in the size of macroconidia or width of vesicles among isolates of each species of *C. pseudoreteaudii*, *C. hongkongensis*, and *C. aconidialis* were observed (Table 5). For example, the macroconidia of *C. pseudoreteaudii* isolates CSF15985, CSF16016, CSF16018, and CSF16027 were relatively much shorter than those of the other three tested *C. pseudoreteaudii* isolates CSF16056, CSF16102 and CSF16116 (Table 5); the vesicles of *C. hongkongensis* isolates CSF16145 and CSF16237 were much wider than those of isolates CSF16121 and CSF16230 (Table 5); in *C. aconidialis*, the vesicles of isolate CSF16130 were much longer than those of isolate CSF16131 (Table 5). The average of measurements further showed that no significant variations also exist in the size of macroconidia or width of vesicles among all isolates of each species of *C. pseudoreteaudii*, *C. hongkongensis*, or *C. aconidialis* were observed (Table 5). For example, the width of vesicles among seven isolates of *C. pseudoreteaudii* were similar, and there were no major differences in macroconidia size among isolates of *C. hongkongensis* and *C. aconidialis* (Table 5).

For each of the five *Calonectria* species found in this study, the shape of vesicle and septate number of macroconidia among isolates obtained during the current study and the originally described strains were consistent (Table 5). The measurements showed that the macroconidia size and vesicles width of isolates obtained in the current study and the originally described strains of the same *Calonectria* species were not always similar; for example, the macroconidia lengths of *C. pseudoreteaudii* isolates obtained in this study were much shorter than the originally described strains of *C. pseudoreteaudii* [30], and the macroconidia length of *C. hongkongensis* isolates obtained in the current study was shorter than the originally described *C. hongkongensis* strains [43] (Table 5). For each species of *C. reteaudii* and *C. auriculiformis*, the macroconidia size of isolates obtained in the current study were similar to the originally described strains of relative species [36,44] (Table 5). The vesicle measurements showed that the vesicle widths of *C. hongkongensis* isolates obtained in the current study were much shorter than those of the originally described strains of *C. hongkongensis* [43], while for *C. pseudoreteaudii*, *C. reteaudii* and *C. auriculiformis*, the vesicle widths of isolates obtained in the current study were similar to the originally described strains of relative species [30,36,44] (Table 5).

**Table 5:** Morphological comparisons of *Calonectria* isolates and species obtained in the current study.

Species	Isolate/Species	Macroconidia (L × W) ^1,2,3^	Macroconidia Average (L × W) ^1,2^	Macroconidia Septation	Vesicle Width ^1,2,3^	Vesicle Width Average ^1^
*C. pseudoreteaudii*	Isolate CSF15985 (this study)	(69–)75.5–85(–88) × (6.5–)7–8(–8.5)	80.5 × 7.5	(3–)5(–6)	(2–)2.5–3.5(–4)	3
	Isolate CSF16016 (this study)	(71–)76–88.5(–98) × (6–)6.5–7.5(–8)	82.5 × 7	(3–)5	(2–)2–3.5(–4)	3
	Isolate CSF16018 (this study)	(75.5–)79–87.5(–94.5) × (6.5–)7–8(–9)	83 × 7.5	5	(2–)2–3.5(–4)	2.5
	Isolate CSF16027 (this study)	(71–)78–89.5(–95) × (6–)6.5–8(–9)	84 × 7.5	5	(2–)2.5–3.5(–4.5)	3
	Isolate CSF16056 (this study)	(77.5–)87–104.5(–112.5) × (6–)7–8(–9)	96 × 7.5	5	(3–)3.5–4.5(–5.5)	4
	Isolate CSF16102 (this study)	(84–)87.5–97.5(–105) × (6–)6.5–8(–10)	92.5 × 7.5	5(–7)	(2–)2.5–3.5(–4)	3
	Isolate CSF16116 (this study)	(76–)84.5–96.5(–104) × (5.5–)7.5–8.5(–10)	90.5 × 8	5	(2–)2.5–4(–4.5)	3
	Species (this study)	(69–)79–95(–112.5) × (5.5–)7–8(–10)	87 × 7.5	(3–)5(–7)	(2–)2.5–4(–5.5)	3
	Species [30]	(88–)96–112(–119) × 7–9(–10)	104 × 8	5(–8)	3–5	N/A ^4^
*C. reteaudii*	Isolate CSF16105 (this study)	(71.5–)77.5–87.5(–92.5) × (6–)6.5–7.5(–8)	82.5 × 7	5	(3.5–)4–5(–6)	4.5
	Species [44]	(50–)75–95(–120) × (5–)6–7	84 × 6.5	(1–)5(–6)	(3–)5(–6)	N/A
*C. auriculiformis*	Isolate CSF16185 (this study)	(34–)36.5–43.5(–47) × (3.5–)4–5(–5.5)	40 × 4.5	1	(4.5–)6–9.5(–12)	7.5
	Species [36]	(40–)41–45(–47) × (3–)4–5	43 × 4	1	6–12	N/A
*C. hongkongensis*	Isolate CSF16121 (this study)	(34.5–)36.5–41(–46) × (3.5–)3.5–4.5(–4.5)	39 × 4	1	(3–)3–6(–12)	4.5
	Isolate CSF16145 (this study)	(36–)37–40(–42.5) × (4–)4–4.5(–5)	38.5 × 4.5	1	(5–)5.5–8.5(–10.5)	7
	Isolate CSF16230 (this study)	(35–)37–40.5(–43.5) × (4–)4–4.5(–5)	38.5 × 4.5	1	(3–)3.5–5(–5.5)	4
	Isolate CSF16237 (this study)	(35–)37–41.5(–43) × (3.5–)4–4.5(–5)	39 × 4	1	(3.5–)4.5–8(–11)	6.5
	Species (this study)	(34.5–)36.5–41(–46) × (3.5–)4–4.5(–5)	40 × 4	1	(3–)3.5–6.5(–12)	5
	Species [43]	(38–)45–48(–53) × 4(–4.5)	46.5 × 4	1	8–14	N/A
*C. aconidialis*	Isolate CSF16130 (this study)	(35–)40–46.5(–50.5) × (4–)4–5(–5.5)	43 × 4.5	1	(5–)5.5–9.5(–13)	7.5
	Isolate CSF16131 (this study)	(37–)42–48.5(–53) × (4–)5–5.5(–6)	45.5 × 5.5	1	(3–)2.5–6(–12)	4.5
	Isolate CSF16133 (this study)	(37–)41.5–47(–51) × (3.5–)4–5(–5.5)	44.5 × 4.5	1	(3.5–)4.5–7.5(–10)	6
	Species (this study)	(35–)41–47.5(–53) × (3.5–)4–5.5(–6)	44 × 5	1	(3–)4–8(–13)	6
	Species [29]	N/A	N/A	N/A	N/A	N/A

^1^ All measurements are in µm. ^2^ L × W = length × width. ^3^ Measurements are presented in the format [(minimum–) (average – standard deviation) – (average + standard deviation) (–maximum)]. ^4^ N/A represents data that is not available.

### 3.6. MAT Gene Amplification and Mating Type Assignment

**Figure 3:**
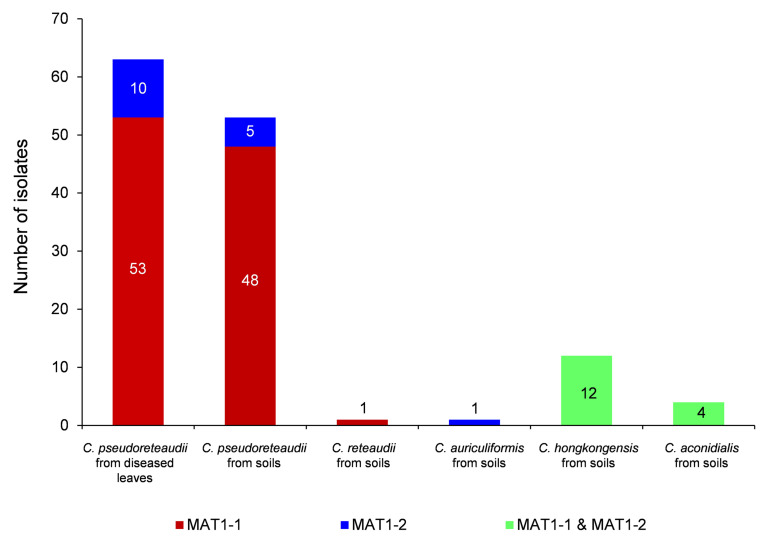
Bar graph showing the distribution of mating type idiomorphs (MAT1-1, MAT1-2, and MAT1-1 and MAT1-2) in five species of *Calonectria*.

Only *C. pseudoreteaudii* was isolated from both diseased leaves and soils under the sampled trees, to understand the differences of mating type assignment between isoaltes from diseased leaves and soils, all the *C. pseudoreteaudii* isolates were selected for *MAT* gene amplification. All the isolates identified as *C. reteaudii*, *C. auriculiformis* and *C. aconidialis* were used for *MAT* gene amplification, since only six isolates were identified as these species. Few isoaltes of *C. hongkongensis* were selected for amplification, since both the *MAT1-1-1* and *MAT1-2-1* genes were successfully amplified for all the selected isolate during the the preliminary *MAT* gene amplification. One hundred and thirty-four *Calonectria* isolates were selected to amplify the *MAT1-1-1* and *MAT1-2-1* genes. These included all 116 isolates of *C. pseudoreteaudii* sequenced for multiple-gene sequence identification (63 isolates from diseased leaves of all ten sampled *Eucalyptus* genotypes, 53 isolates from soils under nine *Eucalyptus* genotypes), 12 random selected isolates of *C. hongkongensis*, and all the six isolates of *C. reteaudii* (CSF16105), *C. auriculiformis* (CSF16185) and *C. aconidialis* (CSF16130, CSF16131, CSF16132 and CSF16133) obtained in the current study (Appendix A Table A1, Figure 3). The mating type idiomorphs were successfully amplified in all 134 *Calonectria* isolates (Appendix A Table A1). Each isolate was identified by positive amplification of a 350 bp fragment *MAT1-1-1* and/or a 270 bp *MAT1-2-1* product. Only the *MAT1-1-1* or *MAT1-2-1* gene was successfully amplified for each isolate of *C. pseudoreteaudii* confirming the heterothallic nature of the species (Figure 3). *Calonectria reteaudii* isolate CSF16105 had only the MAT1-1 mating type, and *C. auriculiformis* isolate CSF16185 had only the MAT1-2 mating type (Figure 3), suggesting that both *C. reteaudii* and *C. auriculiformis* are likely to be heterothallic species. All the amplified isolates of *C. hongkongensis* and *C. aconidialis* had both the MAT1-1 and MAT1-2 mating types, confirming that they are homothallic species (Figure 3).

When considering the mating gene diversity of each *Calonectria* species, only one genotype of *MAT1-1-1* gene was successfully amplified for 101 (53 from diseased leaves, 48 from soils) of the 116 *C. pseudoreteaudii* isolates, two genotypes of *MAT1-2-1* gene for ten isolates from diseased leaves, and one genotype of *MAT1-2-1* gene for five isolates from soil (this genotype is the same as one of the two genotypes from diseased leaves) (Appendix A Table A1, Figure 3). Two genotypes for each of *MAT1-1-1* and *MAT1-2-1* genes were amplified for 12 *C. hongkongensis* isolates from soils (Appendix A Table A1, Figure 3). For the four *C. aconidialis* isolates, only one genotype for each of the *MAT1-1-1* and *MAT1-2-1* genes was amplified (Appendix A Table A1, Figure 3).

For the three heterothallic or putative heterothallic species, *C. pseudoreteaudii*, *C. reteaudii* and *C. auriculiformis*, only *C. pseudoreteaudii* had an adequate number of isolates as a population to test whether recombination took place. The mating type frequencies of MAT1-1 and MAT1-2 for the *C. pseudoreteaudii* isolates from diseased leaves (63 isolates), soils under *Eucalyptus* trees (53 isolates), and from diseased leaves and soils (116 isolates) were 5.3, 9.6 and 6.7, respectively, which all significantly deviated from a 1:1 ratio (*p* < 0.01), implying that the asexual cycle represents the primary reproductive mode in *C. pseudoreteaudii* in the sampled area in the current study (Appendix A Table A1, Figure 3).

### 3.7. Pathogenicity Tests

**Figure 4:**
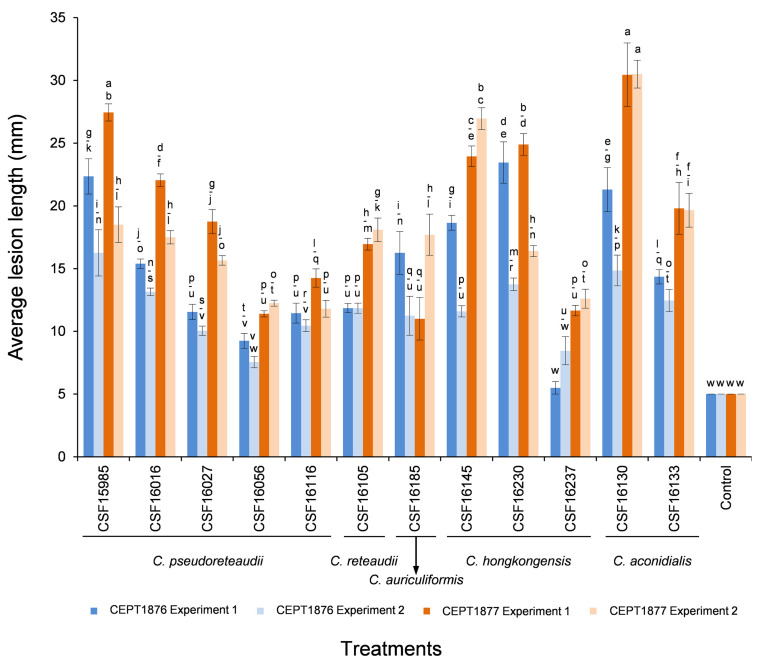
Column chart indicating the average lesion length (mm) on leaves resulting from mycelia plug inoculation trials of two *Eucalyptus* hybrid genotypes inoculated with five *Calonectria* species and the controls; two experiments were conducted. Vertical bars represent standard error of means. Bars topped with different letters indicate treatment means that are significantly different (*p* = 0.05).

Twelve isolates representing five *Calonectria* species (*C. pseudoreteaudii*: CSF15985, CSF16016, CSF16027, CSF16056 and CSF16116; *C. reteaudii*: CSF16105; *C. auriculiformis*: CSF16185; *C. hongkongensis*: CSF16145, CSF16230 and CSF16237; and *C. aconidialis*: CSF16130 and CSF16133) were selected to inoculate on seedlings of two *Eucalyptus* genotypes CEPT1876 and CEPT1877 using mycelia plugs (Appendix A Table A1, Figure 4).

**Figure 5:**
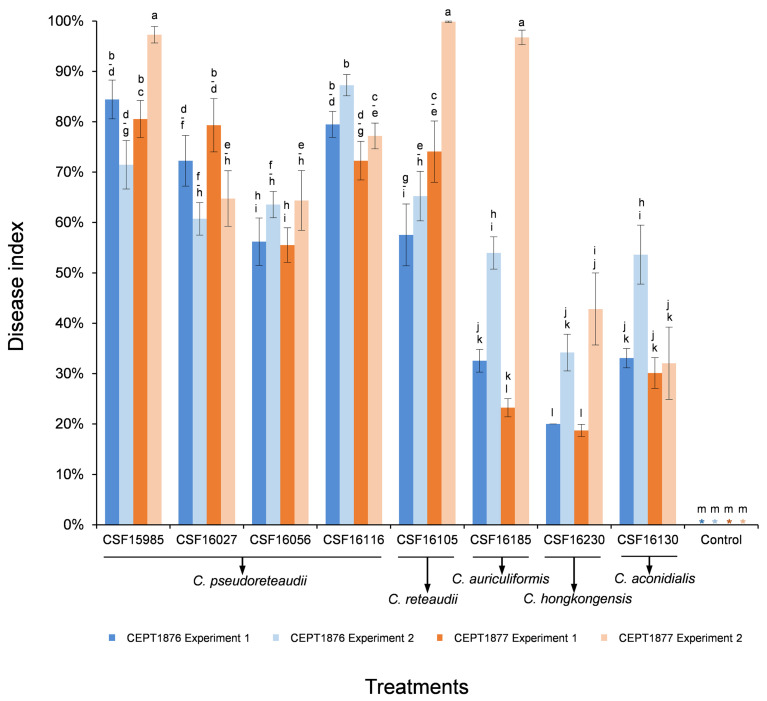
Column chart indicating the disease index (%) resulting from conidia suspension inoculation trials of two *Eucalyptus* hybrid genotypes inoculated with five *Calonectria* species and the controls, two experiments were conducted. Vertical bars represent standard error of means. Bars topped with different letters indicate that treatment means are significantly different (*p* = 0.05). The “*” indicates that the disease indexes of negative controls are zero.

For conidia suspension inoculations, eight isolates representing five *Calonectria* species (*C. pseudoreteaudii*: CSF15985, CSF16027, CSF16056 and CSF16116; *C. reteaudii*: CSF16105; *C. auriculiformis*: CSF16185; *C. hongkongensis*: CSF16230; and *C. aconidialis*: CSF16130) that produced abundant masses of conidiophores and macroconidia were selected to inoculate on seedlings of the two *Eucalyptus* genotypes (Appendix A Table A1, Figure 5).

**Figure 6:**
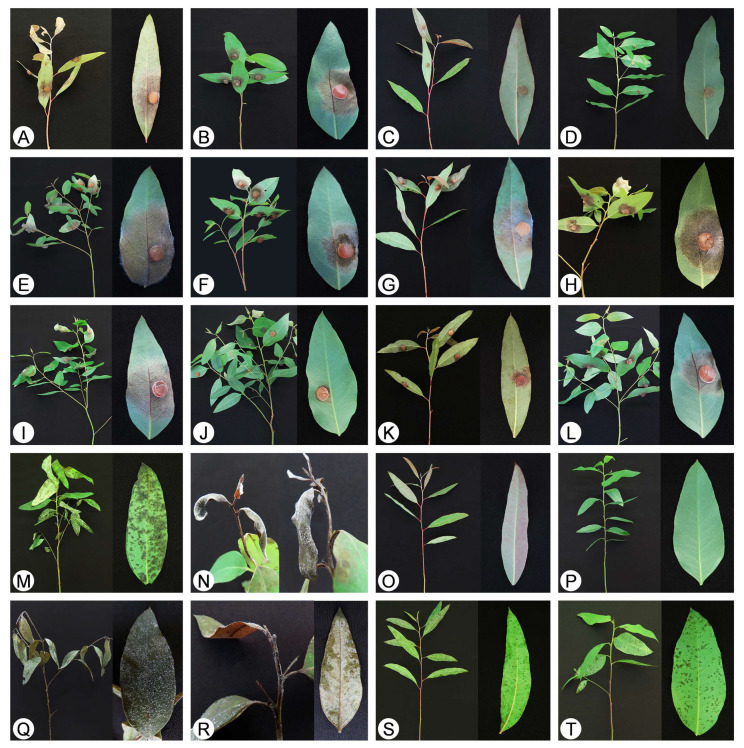
Symptoms on seedlings of *E. urophylla* × *E. tereticornis* hybrid genotype CEPT1876 and *E. urophylla* × *E. grandis* hybrid genotype CEPT1877 inoculated by *Calonectria* mycelia plugs/MEA plugs (**A**–**L**) and conidia suspensions/sterile water (**M**–**T**) of five *Calonectria* isolates. (**A**): CEPT1876 inoculated by isolate CSF16130 (*C. aconidialis*); (**B**): CEPT1877 inoculated by isolate CSF16016 (*C. pseudoreteaudii*); (**C**,**D**): No disease symptoms were observed on leaves of CEPT1876 (**C**) and CEPT1877 (**D**) inoculated by sterile MEA plugs (negative controls); (**E**): Isolate CSF16130 (*C. aconidialis*) produced the longest lesions on CEPT1877; (**F**): Long lesions produced by isolate CSF16145 (*C. hongkongensis*) on CEPT1877; (**G**,**H**): Isolate CSF16230 (*C. hongkongensis*) produced long lesions on CEPT1876 (**G**) and CEPT1877 (**H**); (**I**,**J**): Isolate CSF15985 (*C. pseudoreteaudii*) produced significantly larger lesions on CEPT1877 than that of isolate CSF16056 (*C. pseudoreteaudii*); (**K**,**L**): The inoculation results of isolate CSF16016 (*C. pseudoreteaudii*) indicated that CEPT1876 was significantly more tolerant than CEPT1877; (**M**): Lesions on leaves of CEPT1877 inoculated by isolate CSF16105 (*C. reteaudii*); (**N**): Genotype CEPT1877 rotted after inoculations by isolate CSF16130 (*C. aconidialis*), abundant white mass of conidiophores were observed; (**O**,**P**): No disease symptoms on CEPT1876 (**O**) and CEPT1877 (**P**) inoculated by sterile water (negative controls); (**Q**,**R**): All leaves of CEPT1877 blighted and the seedling died after infection by isolates CSF15985 (**Q**) (*C. pseudoreteaudii*) and CSF16185 (**R**) (*C. auriculiformis*); (**S**,**T**): Lesions on CEPT1876 (**S**) and CEPT1877 (**T**) inoculated by isolate CSF16130 (*C. aconidialis*). (**A**–**L**,**M**–**P**,**S**,**T**) are in the first experiment; (**Q**,**R**) are in the second experiment.

The mycelia plugs of all 12 tested isolates produced leaf spot/lesion symptoms on leaves (Figure 6A,B), while no lesions were observed on the negative control inoculations (Figure 6C,D). All seedlings of the two *Eucalyptus* genotypes inoculated with the conidia suspension of each of the eight *Calonectria* isolates developed leaf spot and shoot blight symptoms (Figure 6M,N), while no disease symptoms were observed on the leaves and shoots of the negative control seedlings (Figure 6O,P). The *Calonectria* species with the same morphological characteristics as the originally inoculated fungi were successfully re-isolated from diseased tissues on the inoculated leaves, but never from the negative control seedlings, thus fulfilling the requirements of Koch’s postulates.

The data of lesion length and disease index resulting from mycelia plug and conidia suspension were not normally distributed based on a Kolmogorov–Smirnov normality test (*p* < 0.05) in SPSS v. 22.0. Thus, all the data were transformed (Kolmogorov–Smirnov normality test, *p* = 0.2) by conducting a Rank transformation using the statistical package SPSS v. 22.0. There were significant differences (*p* < 0.05) between the results of the two experiments of mycelia plug inoculations. The results of two experiments of conidia suspension inoculations also significant differences (*p* < 0.05). This may be due to inconsistent climatic and seedling conditions during the two experiments for each inoculation of mycelia plugs and conidia suspensions. Therefore, the data of each experiment were separately analyzed.

For the mycelia plug inoculations, the two experiments consistently showed that the lesions produced by most isolates in both experiments were significantly larger than the mycelia plug diameter (*p* < 0.05), with the exception of isolate CSF16056 (*C. pseudoreteaudii*) on *Eucalyptus* genotype CEPT1876 in Experiment Two, and isolate CSF16237 (*C. aconidialis*) on CEPT1876 in both experiments (Figure 4). The analyses of average lesion length showed that the longest lesions were produced by isolate CSF16130 (*C. aconidialis*) on *Eucalyptus* genotype CEPT1877 in both experiments (Figure 4 and Figure 6). Overall, isolates CSF15985 (*C. pseudoreteaudii*), CSF16145, CSF16230 (*C. hongkongensis*), and CSF16130 (*C. aconidialis*) produced relatively larger lesions than other isolates on the two tested *Eucalyptus* in the two experiments (Figure 4 and Figure 6E–I). The results showed that the average lesion length caused by some isolates of the same *Calonectria* species was significantly different; for example, on each of the two *Eucalyptus* genotypes in both experiments, the lesions produced by isolate CSF15985 were significantly larger than those of isolates CSF16056 and CSF16116 (*C. pseudoreteaudii*) (*p* < 0.05) (Figure 6I,J), and isolate CSF16145 produced significantly larger lesions than isolate CSF16237 (*C. hongkongensis*) (*p* < 0.05) (Figure 4). The analysis results showed that there were differences in the susceptibility of the two *Eucalyptus* genotypes to the tested isolates. In the two experiments, the average lesion length caused by all 12 *Calonectria* isolates on *Eucalyptus* genotype CEPT1877 was larger than those on genotype CEPT1876, with the exception of isolate CSF16185 in Experiment One (Figure 4 and Figure 6K,L). The results suggested that *Eucalyptus* genotype CEPT1876 is relatively more tolerant than CEPT1877 to the *Calonectria* species tested in this study.

For the conidia suspension inoculations, the two experiments consistently showed that more than 20% of the leaf area of both *Eucalyptus* genotypes were lesioned or rotted after infection by eight inoculated *Calonectria* isolates, with the exception of isolate CSF16230 in Experiment One (Figure 5). The analyses of average disease index showed that isolates CSF15985 (*C. pseudoreteaudii*), CSF16105 (*C. reteaudii*), and CSF16185 (*C. auriculiformis*) caused relatively severe disease in *Eucalyptus* genotype CEPT1877 in Experiment Two, with lesions covering nearly the entire leaf area (Figure 5 and Figure 6Q,R). Overall, isolates of *C. pseudoreteaudii* and *C. reteaudii* were more pathogenic than *C. hongkongensis* and *C. aconidialis* in both experiments (Figure 5). In the two experiments, the average disease indexes generated from eight *Calonectria* isolates on *Eucalyptus* genotype CEPT1877 were higher than those on genotype CEPT1876, with the exception of isolates CSF15985, CSF16056 (*C. pseudoreteaudii*), CSF16185 (*C. auriculiformis*), and CSF16230 (*C. hongkongensis*) in Experiment One, and isolates CSF16116 (*C. pseudoreteaudii*) and CSF16130 (*C. aconidialis*) in both experiments (Figure 5 and Figure 6S,T).

Judging from both the mycelia plug and conidia suspension inoculations, all the tested isolates of five *Calonectria* species produced diseased spots/lesions on the leaves of two inoculated *Eucalyptus* genotypes within three days; pathogenicity differences existed among isolates of the same *Calonectria* species; and *Eucalyptus* genotype CEPT1876 was relatively more tolerant than CEPT1877 to the majority of *Calonectria* isolates tested in this study (Figure 4 and Figure 5). The results further showed that the relative pathogenicity among five *Calonectria* species were not consistent between the inoculations of mycelia plug and conidia suspension. Overall, the pathogenicities among the five *Calonectria* species in mycelia plug inoculations were similar, while *C. hongkongensis* and *C. aconidialis* were less pathogenic than *C. pseudoreteaudii* and *C. reteaudii* in conidia suspension inoculation (Figure 4 and Figure 5).

## 4. Discussion

In this study, leaf disease with the typical symptoms caused by *Calonectria* species was observed in one *Eucalyptus* experimental plantation in GuangXi Province in southern China. A relatively large number of *Calonectria* isolates were isolated from diseased leaves of ten *Eucalyptus* genotypes and soils under the sampled trees in the plantation. Two hundred and forty-five isolates were identified based on DNA sequence comparisons and combined with the morphological characteristics of representative isolates. These fungi were identified as *C. pseudoreteaudii*, *C. reteaudii*, *C. auriculiformis*, *C. hongkongensis* and *C. aconidialis*. *Calonectria pseudoreteaudii* and *C. hongkongensis* were the dominant species, and this is the first report of *C. reteaudii* and *C. auriculiformis* isolated in China. With the exception of *C. pseudoreteaudii*, which was isolated from both diseased *Eucalyptus* leaves and soils, the other four species were only isolated from soils. For the isolates obtained from soils, *C. hongkongensis* (68% of the isolates from soils) was the dominant species, followed by *C. pseudoreteaudii* (29% of the isolates from soils). The *MAT* genes amplification and mating type frequency test results showed that *C. pseudoreteaudii* is a heterothallic species, and the asexual cycle represents the primary reproductive mode, *C. reteaudii* and *C. auriculiformis* are likely to be heterothallic species, and *C. hongkongensis* and *C. aconidialis* are homothallic species. Inoculations indicated that the five *Calonectria* species were all pathogenic to the two tested *Eucalyptus* genotypes.

The isolates obtained in this study were identified mainly based on DNA sequence comparisons of *tef1*, *tub2*, *cmdA* and *his3* gene regions. The sequences of the four genes have been widely used to clearly distinguish between intra- and inter-specific divergence of the *Calonectria* genus [26,32,36]. Recently, Liu and co-authors [28] conducted a comprehensive phylogenetic analyses of the *Calonectria* genus based on DNA sequences of eight gene regions; the results showed that *tef1* and *tub2* sequences had the strongest ability to correctly identify species, followed by *cmdA*, *his3*, *rpb2* and *act* gene regions, and that these six gene regions are effective DNA barcodes for *Calonectria*. The phylogenetic analyses indicated that *Calonectria* species could be recognized based on the *tef1*, *tub2*, *cmdA* and *his3* gene region phylogeny [28]. The phylogenetic analysis results in this study showed that *C. pseudoreteaudii* and *C. reteaudii* belong the *C. reteaudii* species complex, *C. auriculiformis* in the *C. cylindrospora* species complex, and *C. hongkongensis* and *C. aconidialis* in the *C. kyotensis* species complex. The morphological comparisons in the current study indicated that the vesicle shape and septate number of macroconidia are relatively stable among isolates of the same species, while the morphological overlaps about shape of vesicle and septate number of macroconidia exist between *Calonectria* species in the same species complex (*C. pseudoreteaudii* and *C. reteaudii*; *C. hongkongensis* and *C. aconidialis*). The morphological results further showed that significant variations in macroconidia size or vesicle width exist among *Calonectria* isolates of the same species, which is consistent with the results of previous studies [20]. Results in this study confirmed the importance of multi-gene sequence phylogeny in species clarification and identification in *Calonectria*.

*Calonectria pseudoreteaudii* and *C. hongkongensis* were the two dominant species obtained in this study. *Calonectria pseudoreteaudii* was isolated from diseased leaves of all ten sampled *Eucalyptus* genotypes. Both *C. pseudoreteaudii* and *C. hongkongensis* were isolated from soils under nine of the ten *Eucalyptus* genotypes. *Calonectria pseudoreteaudii* was first isolated from diseased *Eucalyptus* cuttings in one nursery in GuangDong Province in southern China [30]; the fungus was further isolated from diseased *Eucalyptus* leaves in FuJian, GuangDong, GuangXi and HaiNan Provinces [19,28,45], and from soils under plantation *Eucalyptus* trees in HaiNan Province [28,29] and soils under the *Eucalyptus* seedlings in a nursery in GuangDong Province [19,28]. Recently, this species were widely obtained from ten *Eucalyptus* species and a number of *E. grandis*, *E. tereticornis* and *E. urophylla* hybrid genotypes in *Eucalyptus* plantations at 13 sites and one nursery in Leizhou Peninsula in southern China [20,28], and also from *Macadamia* sp. in YunNan Province [46]. The results of previous and current studies indicated that *C. pseudoreteaudii* may be distributed in more geographic regions and more plant hosts, both on plant tissues and in soils. *Calonectria hongkongensis* was first isolated from soil in Hong Kong [43]; currently, this fungus has been isolated from soils under the *Eucalyptus* trees in GuangXi and GuangDong and HaiNan Provinces [19,28,29], and from soils under natural forests in FuJian Province and Hong Kong [19,28]. No *C. hongkongensis* has been isolated from diseased tissues till now, which indicated that this species may be widely distributed in soils in different geographic regions in southern China.

Only one to four isolates were obtained for each species of *C. reteaudii*, *C. auriculiformis* and *C. aconidialis* in this study. *Calonectria reteaudii* has been reported from diseased leaves of multiple *Eucalyptus* species in Vietnam [23,28,36]. *Calonectria auriculiformis* was first reported from soils in an *Acacia auriculiformis* plantation in Vietnam [28,36]. Both *C. reteaudii* and *C. auriculiformis* have never been reported in China until now. *Calonectria aconidialis* was first isolated and described from soils under *Eucalyptus* plantations in GuangDong, GuangXi and HaiNan Provinces in southern China [29], and was later reported from other regions in GuangXi Province [19]. Combined with the results of previous research and current studies, *C. reteaudii* may cause disease in *Eucalyptus* trees in China, and the three species are likely distributed in soils in other un-sampled areas in southern China.

Previous research results showed that *C. pseudoreteaudii* [30,35] and *C. reteaudii* [23,44] are heterothallic species, *C. auriculiformis* is probably to be heterothallic [35,36], and both *C. hongkongensis* [35,43] and *C. aconidialis* [29] are homothallic species. Based on the results of *MAT1-1-1* and *MAT1-2-1* gene amplifications, the sexual thallism in *Calonectria* species was successfully detected in Li and co-authors [35]. The detection of the sexual thallism based on *MAT* gene amplifications in five *Calonectria* species in this study is supported by the results of previous studies [30,35,43].

*Calonectria pseudoreteaudii* was frequently isolated from diseased tissues of *Eucalyptus* trees and seedlings widely distributed in southern China [19,20,28], and occasionally in the soils under *Eucalyptus* trees and seedlings [19,29]. The results of the current study indicate that *C. pseudoreteaudii* is widely distributed on both diseased *Eucalyptus* leaves and soils in the plantation. Based on the sequences of *tef1*, *tub2*, *cmdA* and *his3* genes as DNA barcodes and *MAT1-1-1* and *MAT1-2-1* for mating type determination, the genotype diversity of *C. pseudoreteaudii* isolates from diseased leaves is higher than the isolates from soils, and the genotypes generated from diseased leaves contain those from soils. Combined with the research results of the current and previous studies, *C. pseudoreteaudii* is probably propagated from diseased leaves to the soil.

*Calonectria hongkongensis* is the most dominant species isolated in soils in this study. This species was also frequently isolated from soils in other areas in southern China [19,29,43]. For the *C. hongkongensis* isolates obtained in the current study, multiple genotypes were generated based on DNA barcodes genes (six genotypes) and *MAT1-1-1* (two genotypes) and *MAT1-2-1* (two genotypes) genes. No *C. hongkongensis* was identified in diseased *Eucalyptus* leaves both in this and previous studies. Therefore, probably *C. hongkongensis* is a soil-borne species that exclusively inhabits the soil.

The results of pathogenicity tests based on mycelia plug and conidia suspension inoculations in this study showed that all tested isolates of five *Calonectria* species are pathogenic to the two tested *Eucalyptus* genotypes. This was unsurprising for *C. pseudoreteaudii* and *C. reteaudii*, since inoculations in previous studies indicated that *C. pseudoreteaudii* is highly pathogenic to tested *Eucalyptus* genotypes [20], and *C. reteaudii* is considered to be an important pathogen in *Eucalyptus*, as well as many other plants [23]. This study conducted the first pathogenicity test for *C. auriculiformis*, *C. hongkongensis* and *C. aconidialis*. All three species are pathogenic to the *Eucalyptus* genotypes tested, which is cause for concern due to their potential threat to *Eucalyptus*.

Conidia suspensions have been widely used to test the pathogenicities of *Calonectria* species [20,47,48]. However, it is difficult to induce enough conidia to use for inoculations for some isolates/species of *Calonectria*. The pathogenicity test results in the current study showed that relative pathogenicities among five *Calonectria* species are not consistent between the inoculations of mycelia plug and conidia suspension. The conidia need to germinate to develop appressoria and mycelia that penetrate plant cells; the disease symptoms of inoculations using conidia took longer to be observed, which is one of the potential reasons the results of two inoculations were not consistent.

This study expanded our understanding of the species diversity, morphological characteristics, host/substrate range, mating strategy, mating type assignment, genetic diversity and pathogenicity of *Calonectria* species in diseased leaves and soils in the same *Eucalyptus* plantation. Results indicated that there were differences in diversity and host/substrate range among species from diseased leaves and soils, differences in genetic diversity between isolates of the same species from diseased leaves and soils, and some pathogenic species were only isolated from soils but never from diseased leaves. Further studies are necessary to increase the knowledge on fungi ecological niche, the propagation pathway for these species, and the pathogenesis of these species. The inoculation results further indicated that the tolerance of different *Eucalyptus* genotypes are different, which highlights the importance of selecting disease resistant *Eucalyptus* genotypes in the future.

## Figures and Tables

**Table 2:** Samples and isolates of *Calonectria* obtained from ten *Eucalyptus* genotypes in this study.

*Eucalyptus* Genotype	Samples and Isolates from Diseased Leaves of *Eucalyptus* Trees			Samples and Isolates from Soil under *Eucalyptus* Trees			Number of Isolates in Total ^3^
	Number of Samples	Number of Samples Obtained *Calonectria*	Number of *Calonectria* Isolates Obtained ^1^	Number of Samples	Number of Samples Obtained *Calonectria*	Number of *Calonectria* Isolates Obtained ^2^	
CEPT1860	20	20	20	20	5	17	37
CEPT1861	20	20	20	20	5	22	42
CEPT1862	13	12	12	13	2	6	18
CEPT1863	17	14	14	17	0	0	14
CEPT1864	20	20	20	20	4	19	39
CEPT1865	20	19	21	20	5	18	39
CEPT1866	20	19	19	20	6	23	42
CEPT1867	20	20	20	20	6	24	44
CEPT1868	20	20	20	20	12	47	67
CEPT1869	20	20	20	20	2	6	26
In total	190	184	186	190	47	182	368

^1^ One isolate obtained from each sampled tree, with the exception of CEPT1865. ^2^ One to six isolates obtained from each sampled soil. ^3^ The number of isolates obtained from diseased leaves and soils associated with each *Eucalyptus* genotype.

**Table 3:** Statistical values of datasets for maximum parsimony and maximum likelihood analyses in this study.

**Dataset**	**No. of Taxa**	**No. of bp ^1^**	**Maximum Parsimony**						
			**PIC ^2^**	**No. of Trees**	**Tree Length**	**CI ^3^**	**RI ^4^**	**RC ^5^**	**HI ^6^**
*tef1*	118	541	248	211	699	0.635	0.967	0.614	0.365
*tub2*	100	603	273	32	732	0.653	0.956	0.624	0.347
*cmdA*	117	696	277	574	614	0.671	0.974	0.654	0.329
*his3*	115	467	177	1000	716	0.520	0.937	0.487	0.480
*tef1/tub2/cmdA/his3*	118	2307	976	60	2948	0.579	0.955	0.553	0.421
**Dataset**	**Maximum Likelihood**								
	**Subst. Mode ^7^**	**NST ^8^**	**Rate Matrix**					**Rates**	
*tef1*	TIM2+G	6	1.8702	3.2374	1.8702	1.0000	4.9593	Gamma	
*tub2*	TPM3uf+I+G	6	1.3590	4.3533	1.0000	1.3590	4.3533	Gamma	
*cmdA*	TIM1+G	6	1.0000	3.7676	0.7408	0.7408	6.0281	Gamma	
*his3*	TIM3+I+G	6	0.5829	3.0880	1.0000	0.5829	4.1580	Gamma	
*tef1/tub2/cmdA/his3*	TIM2+I+G	6	1.4653	4.3514	1.4653	1.0000	5.3504	Gamma	

^1^ bp = base pairs. ^2^ PIC = number of parsimony informative characters. ^3^ CI = consistency index. ^4^ RI = retention index. ^5^ RC = rescaled consistency index. ^6^ HI = homoplasy index. ^7^ Subst. model = best fit substitution model. ^8^ NST = number of substitution rate categories.

**Table 4:** Species and genetic diversity of *Calonectria* associated with *Eucalyptus* genotype and substrate.

*Eucalyptus* Genotype	*Calonectria* from Diseased Leaves	*Calonectria* from Soils				
	Genotype of *C. pseudoreteaudii*	Genotype of *C. pseudoreteaudii*	Genotype of *C. reteaudii*	Genotype of *C. auriculiformis*	Genotype of *C. hongkongensis*	Genotype of *C. aconidialis*
CEPT1860	GT1 ^1^	GT1	No ^2^	No	GT1	No
CEPT1861	GT1	GT1	No	No	GT1	No
CEPT1862	GT1	GT1	No	No	GT5	No
CEPT1863	GT1	No	No	No	No	No
CEPT1864	GT1	GT1	GT1	No	GT1 and GT2	No
CEPT1865	GT1	GT1	No	No	GT1 and GT3	GT1
CEPT1866	GT1	GT1	No	No	GT1 and GT6	No
CEPT1867	GT1	GT1	No	No	GT1	No
CEPT1868	GT1 and GT2	GT1	No	GT1	GT1, GT2 and GT4	No
CEPT1869	GT1	GT1	No	No	GT1	No

^1^ “GT1”, “GT2”, “GT3”, “GT4”, “GT5” and “GT6” mean Genotype 1, Genotype 2, Genotype 3, Genotype 4, Genotype 5 and Genotype 6, respectively. ^2^ “No” means no *Calonectria* isolate was obtained.

## Data Availability

Data is contained within the article and Supplementary Materials.

## Supplementary Materials

The following are available online at https://www.mdpi.com/2309-608X/7/2/73/s1, Figure S1: Phylogenetic tree of Calonectria species based on maximum likelihood (ML) analyses of the *tef1* gene sequences, Figure S2: Phylogenetic tree of Calonectria species based on ML analyses of the *tub2* gene sequences, Figure S3: Phylogenetic tree of Calonectria species based on ML analyses of the *cmdA* gene sequences, Figure S4: Phylogenetic tree of Calonectria species based on ML analyses of the *his3* gene sequences.

## Appendix A

**Table A1:** Isolates sequenced and used for phylogenetic analyses, mating studies, morphological studies and pathogenicity tests in this study.

Identity	Genotype ^1^	Isolate No. ^2^	Sample No.	Host/Substrate	Collectors	Mating Gene Genotype ^3^		Thallism ^4^	Mating Type	GenBank Accession No. ^5^					
						*MAT1-1-1*	*MAT1-2-1*			*MAT1-1-1*	*MAT1-2-1*	*tef1*	*tub2*	*cmdA*	*his3*
*C. pseudoreteaudii*	AA--	CSF15865 ^6^	20181021-1-(9)	*Eucalyptus* genotype CEPT1860	S.F. Chen, G.Q. Li & Q.C. Wang	MAT1-1-1_GT1	No ^7^	HE	MAT1-1	MW290683	No	MW285158	MW285398	– ^8^	–
*C. pseudoreteaudii*	AAAA	CSF15866 ^6,9^	20181021-1-(11)	*Eucalyptus* genotype CEPT1860	S.F. Chen, G.Q. Li & Q.C. Wang	MAT1-1-1_GT1	No	HE	MAT1-1	MW290684	No	MW285159	MW285399	MW290541	MW290611
*C. pseudoreteaudii*	AA--	CSF15877 ^6^	20181021-1-(33)	*Eucalyptus* genotype CEPT1860	S.F. Chen, G.Q. Li & Q.C. Wang	MAT1-1-1_GT1	No	HE	MAT1-1	MW290685	No	MW285160	MW285400	–	–
*C. pseudoreteaudii*	AA--	CSF15879 ^6^	20181021-1-(37)	*Eucalyptus* genotype CEPT1860	S.F. Chen, G.Q. Li & Q.C. Wang	MAT1-1-1_GT1	No	HE	MAT1-1	MW290686	No	MW285161	MW285401	–	–
*C. pseudoreteaudii*	AA--	CSF15881 ^6^	20181021-1-(41)	*Eucalyptus* genotype CEPT1861	S.F. Chen, G.Q. Li & Q.C. Wang	MAT1-1-1_GT1	No	HE	MAT1-1	MW290687	No	MW285162	MW285402	–	–
*C. pseudoreteaudii*	AAAA	CSF15882 ^6,9^	20181021-1-(43)	*Eucalyptus* genotype CEPT1861	S.F. Chen, G.Q. Li & Q.C. Wang	MAT1-1-1_GT1	No	HE	MAT1-1	MW290688	No	MW285163	MW285403	MW290542	MW290612
*C. pseudoreteaudii*	AA--	CSF15886 ^6^	20181021-1-(51)	*Eucalyptus* genotype CEPT1861	S.F. Chen, G.Q. Li & Q.C. Wang	No	MAT1-2-1_GT1	HE	MAT1-2	No	MW319732	MW285164	MW285404	–	–
*C. pseudoreteaudii*	AA--	CSF15887 ^6^	20181021-1-(53)	*Eucalyptus* genotype CEPT1861	S.F. Chen, G.Q. Li & Q.C. Wang	MAT1-1-1_GT1	No	HE	MAT1-1	MW290689	No	MW285165	MW285405	–	–
*C. pseudoreteaudii*	AA--	CSF15888 ^6^	20181021-1-(55)	*Eucalyptus* genotype CEPT1861	S.F. Chen, G.Q. Li & Q.C. Wang	MAT1-1-1_GT1	No	HE	MAT1-1	MW290690	No	MW285166	MW285406	–	–
*C. pseudoreteaudii*	AA--	CSF15892 ^6^	20181021-1-(63)	*Eucalyptus* genotype CEPT1861	S.F. Chen, G.Q. Li & Q.C. Wang	MAT1-1-1_GT1	No	HE	MAT1-1	MW290691	No	MW285167	MW285407	–	–
*C. pseudoreteaudii*	AA--	CSF15901 ^6^	20181021-1-(81)	*Eucalyptus* genotype CEPT1862	S.F. Chen, G.Q. Li & Q.C. Wang	MAT1-1-1_GT1	No	HE	MAT1-1	MW290692	No	MW285168	MW285408	–	–
*C. pseudoreteaudii*	AAAA	CSF15903 ^6,9^	20181021-1-(85)	*Eucalyptus* genotype CEPT1862	S.F. Chen, G.Q. Li & Q.C. Wang	MAT1-1-1_GT1	No	HE	MAT1-1	MW290693	No	MW285169	MW285409	MW290543	MW290613
*C. pseudoreteaudii*	AA--	CSF15906 ^6^	20181021-1-(91)	*Eucalyptus* genotype CEPT1862	S.F. Chen, G.Q. Li & Q.C. Wang	MAT1-1-1_GT1	No	HE	MAT1-1	MW290694	No	MW285170	MW285410	–	–
*C. pseudoreteaudii*	AA--	CSF15908 ^6^	20181021-1-(95)	*Eucalyptus* genotype CEPT1862	S.F. Chen, G.Q. Li & Q.C. Wang	MAT1-1-1_GT1	No	HE	MAT1-1	MW290695	No	MW285171	MW285411	–	–
*C. pseudoreteaudii*	AA--	CSF15912 ^6^	20181021-1-(105)	*Eucalyptus* genotype CEPT1862	S.F. Chen, G.Q. Li & Q.C. Wang	MAT1-1-1_GT1	No	HE	MAT1-1	MW290696	No	MW285172	MW285412	–	–
*C. pseudoreteaudii*	AAAA	CSF15913 ^6,9^	20181021-1-(107)	*Eucalyptus* genotype CEPT1863	S.F. Chen, G.Q. Li & Q.C. Wang	No	MAT1-2-1_GT1	HE	MAT1-2	No	MW319733	MW285173	MW285413	MW290544	MW290614
*C. pseudoreteaudii*	AA--	CSF15914 ^6^	20181021-1-(109)	*Eucalyptus* genotype CEPT1863	S.F. Chen, G.Q. Li & Q.C. Wang	MAT1-1-1_GT1	No	HE	MAT1-1	MW290697	No	MW285174	MW285414	–	–
*C. pseudoreteaudii*	AA--	CSF15916 ^6^	20181021-1-(113)	*Eucalyptus* genotype CEPT1863	S.F. Chen, G.Q. Li & Q.C. Wang	MAT1-1-1_GT1	No	HE	MAT1-1	MW290698	No	MW285175	MW285415	–	–
*C. pseudoreteaudii*	-A--	CSF15919 ^6^	20181021-1-(119)	*Eucalyptus* genotype CEPT1863	S.F. Chen, G.Q. Li & Q.C. Wang	No	MAT1-2-1_GT1	HE	MAT1-2	No	MW319734	No	MW285416	–	–
*C. pseudoreteaudii*	AA--	CSF15922 ^6^	20181021-1-(129)	*Eucalyptus* genotype CEPT1863	S.F. Chen, G.Q. Li & Q.C. Wang	MAT1-1-1_GT1	No	HE	MAT1-1	MW290699	No	MW285176	MW285417	–	–
*C. pseudoreteaudii*	AA--	CSF15925 ^6^	20181021-1-(137)	*Eucalyptus* genotype CEPT1863	S.F. Chen, G.Q. Li & Q.C. Wang	MAT1-1-1_GT1	No	HE	MAT1-1	MW290700	No	MW285177	MW285418	–	–
*C. pseudoreteaudii*	AAAA	CSF15927 ^6^	20181021-1-(141)	*Eucalyptus* genotype CEPT1864	S.F. Chen, G.Q. Li & Q.C. Wang	MAT1-1-1_GT1	No	HE	MAT1-1	MW290701	No	MW285178	MW285419	MW290545	MW290615
*C. pseudoreteaudii*	AAAA	CSF15933 ^6^	20181021-1-(153)	*Eucalyptus* genotype CEPT1864	S.F. Chen, G.Q. Li & Q.C. Wang	MAT1-1-1_GT1	No	HE	MAT1-1	MW290702	No	MW285179	MW285420	MW290546	MW290616
*C. pseudoreteaudii*	AAAA	CSF15936 ^6,9^	20181021-1-(159)	*Eucalyptus* genotype CEPT1864	S.F. Chen, G.Q. Li & Q.C. Wang	MAT1-1-1_GT1	No	HE	MAT1-1	MW290703	No	MW285180	MW285421	MW290547	MW290617
*C. pseudoreteaudii*	AA--	CSF15939 ^6^	20181021-1-(165)	*Eucalyptus* genotype CEPT1864	S.F. Chen, G.Q. Li & Q.C. Wang	MAT1-1-1_GT1	No	HE	MAT1-1	MW290704	No	MW285181	MW285422	–	–
*C. pseudoreteaudii*	AA--	CSF15942 ^6^	20181021-1-(171)	*Eucalyptus* genotype CEPT1864	S.F. Chen, G.Q. Li & Q.C. Wang	MAT1-1-1_GT1	No	HE	MAT1-1	MW290705	No	MW285182	MW285423	–	–
*C. pseudoreteaudii*	AA--	CSF15947 ^6^	20181021-1-(181)	*Eucalyptus* genotype CEPT1865	S.F. Chen, G.Q. Li & Q.C. Wang	MAT1-1-1_GT1	No	HE	MAT1-1	MW290706	No	MW285183	MW285424	–	–
*C. pseudoreteaudii*	AAAA	CSF15955 ^6^	20181021-1-(195)	*Eucalyptus* genotype CEPT1865	S.F. Chen, G.Q. Li & Q.C. Wang	MAT1-1-1_GT1	No	HE	MAT1-1	MW290707	No	MW285184	MW285425	MW290548	MW290618
*C. pseudoreteaudii*	AAAA	CSF15956 ^6^	20181021-1-(197)	*Eucalyptus* genotype CEPT1865	S.F. Chen, G.Q. Li & Q.C. Wang	MAT1-1-1_GT1	No	HE	MAT1-1	MW290708	No	MW285185	MW285426	MW290549	MW290619
*C. pseudoreteaudii*	AAAA	CSF15959 ^6,9^	20181021-1-(205)	*Eucalyptus* genotype CEPT1865	S.F. Chen, G.Q. Li & Q.C. Wang	MAT1-1-1_GT1	No	HE	MAT1-1	MW290709	No	MW285186	MW285427	MW290550	MW290620
*C. pseudoreteaudii*	AA--	CSF15964 ^6^	20181021-1-(213)	*Eucalyptus* genotype CEPT1865	S.F. Chen, G.Q. Li & Q.C. Wang	MAT1-1-1_GT1	No	HE	MAT1-1	MW290710	No	MW285187	MW285428	–	–
*C. pseudoreteaudii*	AAAA	CSF15965 ^6^	20181021-1-(215)	*Eucalyptus* genotype CEPT1865	S.F. Chen, G.Q. Li & Q.C. Wang	MAT1-1-1_GT1	No	HE	MAT1-1	MW290711	No	MW285188	MW285429	MW290551	MW290621
*C. pseudoreteaudii*	AAAA	CSF15968 ^6^	20181021-1-(221)	*Eucalyptus* genotype CEPT1866	S.F. Chen, G.Q. Li & Q.C. Wang	MAT1-1-1_GT1	No	HE	MAT1-1	MW290712	No	MW285189	MW285430	MW290552	MW290622
*C. pseudoreteaudii*	-A--	CSF15971 ^6^	20181021-1-(227)	*Eucalyptus* genotype CEPT1866	S.F. Chen, G.Q. Li & Q.C. Wang	MAT1-1-1_GT1	No	HE	MAT1-1	MW290713	No	No	MW285431	–	–
*C. pseudoreteaudii*	AA--	CSF15972 ^6^	20181021-1-(229)	*Eucalyptus* genotype CEPT1866	S.F. Chen, G.Q. Li & Q.C. Wang	MAT1-1-1_GT1	No	HE	MAT1-1	MW290714	No	MW285190	MW285432	–	–
*C. pseudoreteaudii*	AAAA	CSF15985 ^6,9,10,11^	20181021-1-(257)	*Eucalyptus* genotype CEPT1866	S.F. Chen, G.Q. Li & Q.C. Wang	MAT1-1-1_GT1	No	HE	MAT1-1	MW290715	No	MW285191	MW285433	MW290553	MW290623
*C. pseudoreteaudii*	AA--	CSF15986 ^6^	20181021-1-(259)	*Eucalyptus* genotype CEPT1866	S.F. Chen, G.Q. Li & Q.C. Wang	MAT1-1-1_GT1	No	HE	MAT1-1	MW290716	No	MW285192	MW285434	–	–
*C. pseudoreteaudii*	AA--	CSF15987 ^6^	20181021-1-(261)	*Eucalyptus* genotype CEPT1867	S.F. Chen, G.Q. Li & Q.C. Wang	MAT1-1-1_GT1	No	HE	MAT1-1	MW290717	No	MW285193	MW285435	–	–
*C. pseudoreteaudii*	AA--	CSF15991 ^6^	20181021-1-(269)	*Eucalyptus* genotype CEPT1867	S.F. Chen, G.Q. Li & Q.C. Wang	MAT1-1-1_GT1	No	HE	MAT1-1	MW290718	No	MW285194	MW285436	–	–
*C. pseudoreteaudii*	AAAA	CSF15993 ^6,9^	20181021-1-(273)	*Eucalyptus* genotype CEPT1867	S.F. Chen, G.Q. Li & Q.C. Wang	MAT1-1-1_GT1	No	HE	MAT1-1	MW290719	No	MW285195	MW285437	MW290554	MW290624
*C. pseudoreteaudii*	AAAA	CSF15995 ^6^	20181021-1-(277)	*Eucalyptus* genotype CEPT1867	S.F. Chen, G.Q. Li & Q.C. Wang	MAT1-1-1_GT1	No	HE	MAT1-1	MW290720	No	MW285196	MW285438	MW290555	MW290625
*C. pseudoreteaudii*	AA--	CSF15996 ^6^	20181021-1-(279)	*Eucalyptus* genotype CEPT1867	S.F. Chen, G.Q. Li & Q.C. Wang	MAT1-1-1_GT1	No	HE	MAT1-1	MW290721	No	MW285197	MW285439	–	–
*C. pseudoreteaudii*	AA--	CSF15998 ^6^	20181021-1-(283)	*Eucalyptus* genotype CEPT1867	S.F. Chen, G.Q. Li & Q.C. Wang	MAT1-1-1_GT1	No	HE	MAT1-1	MW290722	No	MW285198	MW285440	–	–
*C. pseudoreteaudii*	AA--	CSF15999 ^6^	20181021-1-(285)	*Eucalyptus* genotype CEPT1867	S.F. Chen, G.Q. Li & Q.C. Wang	MAT1-1-1_GT1	No	HE	MAT1-1	MW290723	No	MW285199	MW285441	–	–
*C. pseudoreteaudii*	AA--	CSF16001 ^6^	20181021-1-(289)	*Eucalyptus* genotype CEPT1867	S.F. Chen, G.Q. Li & Q.C. Wang	No	MAT1-2-1_GT1	HE	MAT1-2	No	MW319735	MW285200	MW285442	–	–
*C. pseudoreteaudii*	AAAA	CSF16007 ^6^	20181021-1-(301)	*Eucalyptus* genotype CEPT1868	S.F. Chen, G.Q. Li & Q.C. Wang	No	MAT1-2-1_GT1	HE	MAT1-2	No	MW319736	MW285201	MW285443	MW290556	MW290626
*C. pseudoreteaudii*	AA--	CSF16008 ^6^	20181021-1-(303)	*Eucalyptus* genotype CEPT1868	S.F. Chen, G.Q. Li & Q.C. Wang	No	MAT1-2-1_GT2	HE	MAT1-2	No	MW319737	MW285202	MW285444	–	–
*C. pseudoreteaudii*	AA--	CSF16009 ^6^	20181021-1-(305)	*Eucalyptus* genotype CEPT1868	S.F. Chen, G.Q. Li & Q.C. Wang	No	MAT1-2-1_GT2	HE	MAT1-2	No	MW319738	MW285203	MW285445	–	–
*C. pseudoreteaudii*	AAAA	CSF16010 ^6^	20181021-1-(307)	*Eucalyptus* genotype CEPT1868	S.F. Chen, G.Q. Li & Q.C. Wang	MAT1-1-1_GT1	No	HE	MAT1-1	MW290724	No	MW285204	MW285446	MW290557	MW290627
*C. pseudoreteaudii*	AA--	CSF16013 ^6^	20181021-1-(313)	*Eucalyptus* genotype CEPT1868	S.F. Chen, G.Q. Li & Q.C. Wang	MAT1-1-1_GT1	No	HE	MAT1-1	MW290725	No	MW285205	MW285447	–	–
*C. pseudoreteaudii*	AA--	CSF16017 ^6^	20181021-1-(321)	*Eucalyptus* genotype CEPT1868	S.F. Chen, G.Q. Li & Q.C. Wang	MAT1-1-1_GT1	No	HE	MAT1-1	MW290726	No	MW285207	MW285449	–	–
*C. pseudoreteaudii*	AAAA	CSF16018 ^6,9,10^	20181021-1-(323)	*Eucalyptus* genotype CEPT1868	S.F. Chen, G.Q. Li & Q.C. Wang	MAT1-1-1_GT1	No	HE	MAT1-1	MW290727	No	MW285208	MW285450	MW290559	MW290629
*C. pseudoreteaudii*	AA--	CSF16019 ^6^	20181021-1-(325)	*Eucalyptus* genotype CEPT1868	S.F. Chen, G.Q. Li & Q.C. Wang	No	MAT1-2-1_GT1	HE	MAT1-2	No	MW319740	MW285209	MW285451	–	–
*C. pseudoreteaudii*	AA--	CSF16021 ^6^	20181021-1-(329)	*Eucalyptus* genotype CEPT1868	S.F. Chen, G.Q. Li & Q.C. Wang	MAT1-1-1_GT1	No	HE	MAT1-1	MW290728	No	MW285210	MW285452	–	–
*C. pseudoreteaudii*	AAAA	CSF16023 ^6^	20181021-1-(333)	*Eucalyptus* genotype CEPT1868	S.F. Chen, G.Q. Li & Q.C. Wang	MAT1-1-1_GT1	No	HE	MAT1-1	MW290729	No	MW285211	MW285453	MW290560	MW290630
*C. pseudoreteaudii*	AA--	CSF16024 ^6^	20181021-1-(335)	*Eucalyptus* genotype CEPT1868	S.F. Chen, G.Q. Li & Q.C. Wang	No	MAT1-2-1_GT2	HE	MAT1-2	No	MW319741	MW285212	MW285454	–	–
*C. pseudoreteaudii*	AAAA	CSF16027 ^6,9,10,11^	20181021-1-(341)	*Eucalyptus* genotype CEPT1869	S.F. Chen, G.Q. Li & Q.C. Wang	MAT1-1-1_GT1	No	HE	MAT1-1	MW290730	No	MW285213	MW285455	MW290561	MW290631
*C. pseudoreteaudii*	AAAA	CSF16031 ^6^	20181021-1-(349)	*Eucalyptus* genotype CEPT1869	S.F. Chen, G.Q. Li & Q.C. Wang	MAT1-1-1_GT1	No	HE	MAT1-1	MW290731	No	MW285214	MW285456	MW290562	MW290632
*C. pseudoreteaudii*	AA--	CSF16035 ^6^	20181021-1-(357)	*Eucalyptus* genotype CEPT1869	S.F. Chen, G.Q. Li & Q.C. Wang	MAT1-1-1_GT1	No	HE	MAT1-1	MW290732	No	MW285215	MW285457	–	–
*C. pseudoreteaudii*	AA--	CSF16039 ^6^	20181021-1-(365)	*Eucalyptus* genotype CEPT1869	S.F. Chen, G.Q. Li & Q.C. Wang	MAT1-1-1_GT1	No	HE	MAT1-1	MW290733	No	MW285216	MW285458	–	–
*C. pseudoreteaudii*	AA--	CSF16042 ^6^	20181021-1-(371)	*Eucalyptus* genotype CEPT1869	S.F. Chen, G.Q. Li & Q.C. Wang	MAT1-1-1_GT1	No	HE	MAT1-1	MW290734	No	MW285217	MW285459	–	–
*C. pseudoreteaudii*	AA--	CSF16045 ^6^	20181021-1-(377)	*Eucalyptus* genotype CEPT1869	S.F. Chen, G.Q. Li & Q.C. Wang	MAT1-1-1_GT1	No	HE	MAT1-1	MW290735	No	MW285218	MW285460	–	–
*C. pseudoreteaudii*	AAAA	CSF16053 ^6^	20181021-1-(12)	soil under *Eucalyptus* genotype CEPT 1860	S.F. Chen, G.Q. Li & Q.C. Wang	MAT1-1-1_GT1	No	HE	MAT1-1	MW290737	No	MW285225	MW285467	MW290564	MW290634
*C. pseudoreteaudii*	AA--	CSF16054 ^6^	20181021-1-(12)	soil under *Eucalyptus* genotype CEPT 1860	S.F. Chen, G.Q. Li & Q.C. Wang	MAT1-1-1_GT1	No	HE	MAT1-1	MW290738	No	MW285226	MW285468	–	–
*C. pseudoreteaudii*	AA--	CSF16055 ^6^	20181021-1-(12)	soil under *Eucalyptus* genotype CEPT 1860	S.F. Chen, G.Q. Li & Q.C. Wang	MAT1-1-1_GT1	No	HE	MAT1-1	MW290739	No	MW285227	MW285469	–	–
*C. pseudoreteaudii*	AAAA	CSF16056 ^6,9,10,11^	20181021-1-(12)	soil under *Eucalyptus* genotype CEPT 1860	S.F. Chen, G.Q. Li & Q.C. Wang	MAT1-1-1_GT1	No	HE	MAT1-1	MW290740	No	MW285228	MW285470	MW290565	MW290635
*C. pseudoreteaudii*	AA--	CSF16057 ^6^	20181021-1-(12)	soil under *Eucalyptus* genotype CEPT 1860	S.F. Chen, G.Q. Li & Q.C. Wang	MAT1-1-1_GT1	No	HE	MAT1-1	MW290741	No	MW285229	MW285471	–	–
*C. pseudoreteaudii*	AA--	CSF16062 ^6^	20181021-1-(38)	soil under *Eucalyptus* genotype CEPT 1860	S.F. Chen, G.Q. Li & Q.C. Wang	MAT1-1-1_GT1	No	HE	MAT1-1	MW290742	No	MW285232	MW285474	–	–
*C. pseudoreteaudii*	AA--	CSF16063 ^6^	20181021-1-(38)	soil under *Eucalyptus* genotype CEPT 1860	S.F. Chen, G.Q. Li & Q.C. Wang	MAT1-1-1_GT1	No	HE	MAT1-1	MW290743	No	MW285233	MW285475	–	–
*C. pseudoreteaudii*	AA--	CSF16064 ^6^	20181021-1-(38)	soil under *Eucalyptus* genotype CEPT 1860	S.F. Chen, G.Q. Li & Q.C. Wang	MAT1-1-1_GT1	No	HE	MAT1-1	MW290744	No	MW285234	MW285476	–	–
*C. pseudoreteaudii*	AAAA	CSF16066 ^6,9^	20181021-1-(44)	soil under *Eucalyptus* genotype CEPT1861	S.F. Chen, G.Q. Li & Q.C. Wang	MAT1-1-1_GT1	No	HE	MAT1-1	MW290745	No	MW285236	MW285478	MW290566	MW290636
*C. pseudoreteaudii*	AA--	CSF16067 ^6^	20181021-1-(44)	soil under *Eucalyptus* genotype CEPT1861	S.F. Chen, G.Q. Li & Q.C. Wang	MAT1-1-1_GT1	No	HE	MAT1-1	MW290746	No	MW285237	MW285479	–	–
*C. pseudoreteaudii*	AA--	CSF16068 ^6^	20181021-1-(44)	soil under *Eucalyptus* genotype CEPT1861	S.F. Chen, G.Q. Li & Q.C. Wang	MAT1-1-1_GT1	No	HE	MAT1-1	MW290747	No	MW285238	MW285480	–	–
*C. pseudoreteaudii*	AA--	CSF16072 ^6^	20181021-1-(52)	soil under *Eucalyptus* genotype CEPT1861	S.F. Chen, G.Q. Li & Q.C. Wang	MAT1-1-1_GT1	No	HE	MAT1-1	MW290748	No	MW285242	MW285484	–	–
*C. pseudoreteaudii*	AA--	CSF16076 ^6^	20181021-1-(54)	soil under *Eucalyptus* genotype CEPT1861	S.F. Chen, G.Q. Li & Q.C. Wang	MAT1-1-1_GT1	No	HE	MAT1-1	MW290749	No	MW285246	MW285488	–	–
*C. pseudoreteaudii*	AA--	CSF16077 ^6^	20181021-1-(54)	soil under *Eucalyptus* genotype CEPT1861	S.F. Chen, G.Q. Li & Q.C. Wang	MAT1-1-1_GT1	No	HE	MAT1-1	MW290750	No	MW285247	MW285489	–	–
*C. pseudoreteaudii*	AA--	CSF16078 ^6^	20181021-1-(54)	soil under *Eucalyptus* genotype CEPT1861	S.F. Chen, G.Q. Li & Q.C. Wang	MAT1-1-1_GT1	No	HE	MAT1-1	MW290751	No	MW285248	MW285490	–	–
*C. pseudoreteaudii*	AA--	CSF16079 ^6^	20181021-1-(54)	soil under *Eucalyptus* genotype CEPT1861	S.F. Chen, G.Q. Li & Q.C. Wang	MAT1-1-1_GT1	No	HE	MAT1-1	MW290752	No	MW285249	MW285491	–	–
*C. pseudoreteaudii*	AA--	CSF16080 ^6^	20181021-1-(56)	soil under *Eucalyptus* genotype CEPT1861	S.F. Chen, G.Q. Li & Q.C. Wang	MAT1-1-1_GT1	No	HE	MAT1-1	MW290753	No	MW285250	MW285492	–	–
*C. pseudoreteaudii*	AA--	CSF16082 ^6^	20181021-1-(56)	soil under *Eucalyptus* genotype CEPT1861	S.F. Chen, G.Q. Li & Q.C. Wang	MAT1-1-1_GT1	No	HE	MAT1-1	MW290754	No	MW285251	MW285493	–	–
*C. pseudoreteaudii*	AA--	CSF16083 ^6^	20181021-1-(56)	soil under *Eucalyptus* genotype CEPT1861	S.F. Chen, G.Q. Li & Q.C. Wang	MAT1-1-1_GT1	No	HE	MAT1-1	MW290755	No	MW285252	MW285494	–	–
*C. pseudoreteaudii*	AA--	CSF16084 ^6^	20181021-1-(56)	soil under *Eucalyptus* genotype CEPT1861	S.F. Chen, G.Q. Li & Q.C. Wang	MAT1-1-1_GT1	No	HE	MAT1-1	MW290756	No	MW285253	MW285495	–	–
*C. pseudoreteaudii*	AA--	CSF16085 ^6^	20181021-1-(64)	soil under *Eucalyptus* genotype CEPT1861	S.F. Chen, G.Q. Li & Q.C. Wang	MAT1-1-1_GT1	No	HE	MAT1-1	MW290757	No	MW285254	MW285496	–	–
*C. pseudoreteaudii*	AA--	CSF16086 ^6^	20181021-1-(64)	soil under *Eucalyptus* genotype CEPT1861	S.F. Chen, G.Q. Li & Q.C. Wang	MAT1-1-1_GT1	No	HE	MAT1-1	MW290758	No	MW285255	MW285497	–	–
*C. pseudoreteaudii*	AA--	CSF16087 ^6^	20181021-1-(64)	soil under *Eucalyptus* genotype CEPT1861	S.F. Chen, G.Q. Li & Q.C. Wang	MAT1-1-1_GT1	No	HE	MAT1-1	MW290759	No	MW285256	MW285498	–	–
*C. pseudoreteaudii*	AA--	CSF16089 ^6^	20181021-1-(64)	soil under *Eucalyptus* genotype CEPT1861	S.F. Chen, G.Q. Li & Q.C. Wang	MAT1-1-1_GT1	No	HE	MAT1-1	MW290760	No	MW285257	MW285499	–	–
*C. pseudoreteaudii*	AAAA	CSF16090 ^6,9^	20181021-1-(86)	soil under *Eucalyptus* genotype CEPT1862	S.F. Chen, G.Q. Li & Q.C. Wang	MAT1-1-1_GT1	No	HE	MAT1-1	MW290761	No	MW285258	MW285500	MW290568	MW290638
*C. pseudoreteaudii*	AA--	CSF16094 ^6^	20181021-1-(106)	soil under *Eucalyptus* genotype CEPT1862	S.F. Chen, G.Q. Li & Q.C. Wang	No	MAT1-2-1_GT1	HE	MAT1-2	No	MW319745	MW285262	MW285504	–	–
*C. pseudoreteaudii*	AA--	CSF16095 ^6^	20181021-1-(106)	soil under *Eucalyptus* genotype CEPT1862	S.F. Chen, G.Q. Li & Q.C. Wang	No	MAT1-2-1_GT1	HE	MAT1-2	No	MW319746	MW285263	MW285505	–	–
*C. pseudoreteaudii*	AAAA	CSF16101 ^6,9^	20181021-1-(154)	soil under *Eucalyptus* genotype CEPT1864	S.F. Chen, G.Q. Li & Q.C. Wang	No	MAT1-2-1_GT1	HE	MAT1-2	No	MW319748	MW285269	MW285511	MW290577	MW290647
*C. pseudoreteaudii*	AA--	CSF16102 ^6,10^	20181021-1-(154)	soil under *Eucalyptus* genotype CEPT1864	S.F. Chen, G.Q. Li & Q.C. Wang	No	MAT1-2-1_GT1	HE	MAT1-2	No	MW319749	MW285270	MW285512	–	–
*C. pseudoreteaudii*	AA--	CSF16103 ^6^	20181021-1-(154)	soil under *Eucalyptus* genotype CEPT1864	S.F. Chen, G.Q. Li & Q.C. Wang	No	MAT1-2-1_GT1	HE	MAT1-2	No	MW319750	MW285271	MW285513	–	–
*C. pseudoreteaudii*	AA--	CSF16114 ^6^	20181021-1-(172)	soil under *Eucalyptus* genotype CEPT1864	S.F. Chen, G.Q. Li & Q.C. Wang	MAT1-1-1_GT1	No	HE	MAT1-1	MW290766	No	MW285281	MW285523	–	–
*C. pseudoreteaudii*	AAAA	CSF16116 ^6,9,10,11^	20181021-1-(196)	soil under *Eucalyptus* genotype CEPT1865	S.F. Chen, G.Q. Li & Q.C. Wang	MAT1-1-1_GT1	No	HE	MAT1-1	MW290767	No	MW285283	MW285525	MW290580	MW290650
*C. pseudoreteaudii*	AA--	CSF16117 ^6^	20181021-1-(196)	soil under *Eucalyptus* genotype CEPT1865	S.F. Chen, G.Q. Li & Q.C. Wang	MAT1-1-1_GT1	No	HE	MAT1-1	MW290768	No	MW285284	MW285526	–	–
*C. pseudoreteaudii*	AA--	CSF16118 ^6^	20181021-1-(196)	soil under *Eucalyptus* genotype CEPT1865	S.F. Chen, G.Q. Li & Q.C. Wang	MAT1-1-1_GT1	No	HE	MAT1-1	MW290769	No	MW285285	MW285527	–	–
*C. pseudoreteaudii*	AA--	CSF16119 ^6^	20181021-1-(196)	soil under *Eucalyptus* genotype CEPT1865	S.F. Chen, G.Q. Li & Q.C. Wang	MAT1-1-1_GT1	No	HE	MAT1-1	MW290770	No	MW285286	MW285528	–	–
*C. pseudoreteaudii*	AAAA	CSF16134 ^6,9^	20181021-1-(222)	soil under *Eucalyptus* genotype CEPT1866	S.F. Chen, G.Q. Li & Q.C. Wang	MAT1-1-1_GT1	No	HE	MAT1-1	MW290777	No	MW285301	MW285539	MW290590	MW290660
*C. pseudoreteaudii*	AA--	CSF16135 ^6^	20181021-1-(222)	soil under *Eucalyptus* genotype CEPT1866	S.F. Chen, G.Q. Li & Q.C. Wang	MAT1-1-1_GT1	No	HE	MAT1-1	MW290778	No	MW285302	MW285540	–	–
*C. pseudoreteaudii*	AA--	CSF16136 ^6^	20181021-1-(222)	soil under *Eucalyptus* genotype CEPT1866	S.F. Chen, G.Q. Li & Q.C. Wang	MAT1-1-1_GT1	No	HE	MAT1-1	MW290779	No	MW285303	MW285541	–	–
*C. pseudoreteaudii*	AA--	CSF16137 ^6^	20181021-1-(222)	soil under *Eucalyptus* genotype CEPT1866	S.F. Chen, G.Q. Li & Q.C. Wang	MAT1-1-1_GT1	No	HE	MAT1-1	MW290780	No	MW285304	MW285542	–	–
*C. pseudoreteaudii*	AA--	CSF16138 ^6^	20181021-1-(228)	soil under *Eucalyptus* genotype CEPT1866	S.F. Chen, G.Q. Li & Q.C. Wang	MAT1-1-1_GT1	No	HE	MAT1-1	MW290781	No	MW285305	MW285543	–	–
*C. pseudoreteaudii*	AA--	CSF16139 ^6^	20181021-1-(228)	soil under *Eucalyptus* genotype CEPT1866	S.F. Chen, G.Q. Li & Q.C. Wang	MAT1-1-1_GT1	No	HE	MAT1-1	MW290782	No	MW285306	MW285544	–	–
*C. pseudoreteaudii*	AA--	CSF16140 ^6^	20181021-1-(228)	soil under *Eucalyptus* genotype CEPT1866	S.F. Chen, G.Q. Li & Q.C. Wang	MAT1-1-1_GT1	No	HE	MAT1-1	MW290783	No	MW285307	MW285545	–	–
*C. pseudoreteaudii*	AA--	CSF16141 ^6^	20181021-1-(230)	soil under *Eucalyptus* genotype CEPT1866	S.F. Chen, G.Q. Li & Q.C. Wang	MAT1-1-1_GT1	No	HE	MAT1-1	MW290784	No	MW285308	MW285546	–	–
*C. pseudoreteaudii*	AA--	CSF16142 ^6^	20181021-1-(230)	soil under *Eucalyptus* genotype CEPT1866	S.F. Chen, G.Q. Li & Q.C. Wang	MAT1-1-1_GT1	No	HE	MAT1-1	MW290785	No	MW285309	MW285547	–	–
*C. pseudoreteaudii*	AA--	CSF16143 ^6^	20181021-1-(230)	soil under *Eucalyptus* genotype CEPT1866	S.F. Chen, G.Q. Li & Q.C. Wang	MAT1-1-1_GT1	No	HE	MAT1-1	MW290786	No	MW285310	MW285548	–	–
*C. pseudoreteaudii*	AA--	CSF16144 ^6^	20181021-1-(230)	soil under *Eucalyptus* genotype CEPT1866	S.F. Chen, G.Q. Li & Q.C. Wang	MAT1-1-1_GT1	No	HE	MAT1-1	MW290787	No	MW285311	MW285549	–	–
*C. pseudoreteaudii*	AAAA	CSF16163 ^6,9^	20181021-1-(278)	soil under *Eucalyptus* genotype CEPT1867	S.F. Chen, G.Q. Li & Q.C. Wang	MAT1-1-1_GT1	No	HE	MAT1-1	MW290790	No	MW285328	MW285566	MW290597	MW290667
*C. pseudoreteaudii*	AA--	CSF16164 ^6^	20181021-1-(278)	soil under *Eucalyptus* genotype CEPT1867	S.F. Chen, G.Q. Li & Q.C. Wang	MAT1-1-1_GT1	No	HE	MAT1-1	MW290791	No	MW285329	MW285567	–	–
*C. pseudoreteaudii*	AA--	CSF16165 ^6^	20181021-1-(278)	soil under *Eucalyptus* genotype CEPT1867	S.F. Chen, G.Q. Li & Q.C. Wang	MAT1-1-1_GT1	No	HE	MAT1-1	MW290792	No	MW285330	MW285568	–	–
*C. pseudoreteaudii*	AA-A	CSF16209 ^6^	20181021-1-(320)	soil under *Eucalyptus* genotype CEPT1868	S.F. Chen, G.Q. Li & Q.C. Wang	MAT1-1-1_GT1	No	HE	MAT1-1	MW290793	No	MW285369	MW285610	No	MW290673
*C. pseudoreteaudii*	AAAA	CSF16211 ^6,9^	20181021-1-(320)	soil under *Eucalyptus* genotype CEPT1868	S.F. Chen, G.Q. Li & Q.C. Wang	MAT1-1-1_GT1	No	HE	MAT1-1	MW290794	No	MW285371	MW285612	MW290603	MW290674
*C. pseudoreteaudii*	AAAA	CSF16235 ^6,9^	20181021-1-(342)	soil under *Eucalyptus* genotype CEPT1869	S.F. Chen, G.Q. Li & Q.C. Wang	MAT1-1-1_GT1	No	HE	MAT1-1	MW290798	No	MW285392	MW285632	MW290609	MW290681
*C. pseudoreteaudii*	AA--	CSF16236 ^6^	20181021-1-(342)	soil under *Eucalyptus* genotype CEPT1869	S.F. Chen, G.Q. Li & Q.C. Wang	MAT1-1-1_GT1	No	HE	MAT1-1	MW290799	No	MW285393	MW285633	–	–
*C. pseudoreteaudii*	AABA	CSF16016 ^6,9,10,11^	20181021-1-(319)	*Eucalyptus* genotype CEPT1868	S.F. Chen, G.Q. Li & Q.C. Wang	No	MAT1-2-1_GT2	HE	MAT1-2	No	MW319739	MW285206	MW285448	MW290558	MW290628
*C. reteaudii*	AAAA	CSF16105 ^6,9,10,11^	20181021-1-(154)	soil under *Eucalyptus* genotype CEPT1864	S.F. Chen, G.Q. Li & Q.C. Wang	MAT1-1-1_GT1	No	P_HE	MAT1-1	MW290765	No	MW285272	MW285514	MW290578	MW290648
*C. auriculiformis*	AAAA	CSF16185 ^6,9,10,11^	20181021-1-(302)	soil under *Eucalyptus* genotype CEPT1868	S.F. Chen, G.Q. Li & Q.C. Wang	No	MAT1-2-1_GT1	P_HE	MAT1-2	No	MW319759	MW285349	MW285587	MW290598	MW290669
*C. hongkongensis*	AAAA	CSF16047 ^6,9^	20181021-1-(2)	soil under *Eucalyptus* genotype CEPT1860	S.F. Chen, G.Q. Li & Q.C. Wang	MAT1-1-1_GT1	MAT1-2-1_GT1	HO	homothallic	MW290736	MW319742	MW285219	MW285461	MW290563	MW290633
*C. hongkongensis*	AA--	CSF16048	20181021-1-(2)	soil under *Eucalyptus* genotype CEPT1860	S.F. Chen, G.Q. Li & Q.C. Wang	–	–	–	–	–	–	MW285220	MW285462	–	–
*C. hongkongensis*	AA--	CSF16049	20181021-1-(10)	soil under *Eucalyptus* genotype CEPT1860	S.F. Chen, G.Q. Li & Q.C. Wang	–	–	–	–	–	–	MW285221	MW285463	–	–
*C. hongkongensis*	AA--	CSF16050	20181021-1-(10)	soil under *Eucalyptus* genotype CEPT1860	S.F. Chen, G.Q. Li & Q.C. Wang	–	–	–	–	–	–	MW285222	MW285464	–	–
*C. hongkongensis*	AA--	CSF16051	20181021-1-(10)	soil under *Eucalyptus* genotype CEPT1860	S.F. Chen, G.Q. Li & Q.C. Wang	–	–	–	–	–	–	MW285223	MW285465	–	–
*C. hongkongensis*	AA--	CSF16052	20181021-1-(10)	soil under *Eucalyptus* genotype CEPT1860	S.F. Chen, G.Q. Li & Q.C. Wang	–	–	–	–	–	–	MW285224	MW285466	–	–
*C. hongkongensis*	AA--	CSF16058	20181021-1-(34)	soil under *Eucalyptus* genotype CEPT1860	S.F. Chen, G.Q. Li & Q.C. Wang	–	–	–	–	–	–	MW285230	MW285472	–	–
*C. hongkongensis*	AA--	CSF16059	20181021-1-(34)	soil under *Eucalyptus* genotype CEPT1860	S.F. Chen, G.Q. Li & Q.C. Wang	–	–	–	–	–	–	MW285231	MW285473	–	–
*C. hongkongensis*	AA--	CSF16065	20181021-1-(38)	soil under *Eucalyptus* genotype CEPT1860	S.F. Chen, G.Q. Li & Q.C. Wang	–	–	–	–	–	–	MW285235	MW285477	–	–
*C. hongkongensis*	AAAA	CSF16069 ^9^	20181021-1-(44)	soil under *Eucalyptus* genotype CEPT1861	S.F. Chen, G.Q. Li & Q.C. Wang	–	–	–	–	–	–	MW285239	MW285481	MW290567	MW290637
*C. hongkongensis*	AA--	CSF16070	20181021-1-(44)	soil under *Eucalyptus* genotype CEPT1861	S.F. Chen, G.Q. Li & Q.C. Wang	–	–	–	–	–	–	MW285240	MW285482	–	–
*C. hongkongensis*	AA--	CSF16071	20181021-1-(52)	soil under *Eucalyptus* genotype CEPT1861	S.F. Chen, G.Q. Li & Q.C. Wang	–	–	–	–	–	–	MW285241	MW285483	–	–
*C. hongkongensis*	AA--	CSF16073	20181021-1-(52)	soil under *Eucalyptus* genotype CEPT1861	S.F. Chen, G.Q. Li & Q.C. Wang	–	–	–	–	–	–	MW285243	MW285485	–	–
*C. hongkongensis*	AA--	CSF16074	20181021-1-(52)	soil under *Eucalyptus* genotype CEPT1861	S.F. Chen, G.Q. Li & Q.C. Wang	–	–	–	–	–	–	MW285244	MW285486	–	–
*C. hongkongensis*	AA--	CSF16075	20181021-1-(52)	soil under *Eucalyptus* genotype CEPT1861	S.F. Chen, G.Q. Li & Q.C. Wang	–	–	–	–	–	–	MW285245	MW285487	–	–
*C. hongkongensis*	AAAA	CSF16106^9^	20181021-1-(160)	soil under *Eucalyptus* genotype CEPT1864	S.F. Chen, G.Q. Li & Q.C. Wang	–	–	–	–	–	–	MW285273	MW285515	MW290579	MW290649
*C. hongkongensis*	AA--	CSF16107	20181021-1-(160)	soil under *Eucalyptus* genotype CEPT1864	S.F. Chen, G.Q. Li & Q.C. Wang	–	–	–	–	–	–	MW285274	MW285516	–	–
*C. hongkongensis*	AA--	CSF16108	20181021-1-(160)	soil under *Eucalyptus* genotype CEPT1864	S.F. Chen, G.Q. Li & Q.C. Wang	–	–	–	–	–	–	MW285275	MW285517	–	–
*C. hongkongensis*	AA--	CSF16109	20181021-1-(160)	soil under *Eucalyptus* genotype CEPT1864	S.F. Chen, G.Q. Li & Q.C. Wang	–	–	–	–	–	–	MW285276	MW285518	–	–
*C. hongkongensis*	AA--	CSF16110	20181021-1-(172)	soil under *Eucalyptus* genotype CEPT1864	S.F. Chen, G.Q. Li & Q.C. Wang	–	–	–	–	–	–	MW285277	MW285519	–	–
*C. hongkongensis*	AA--	CSF16111	20181021-1-(172)	soil under *Eucalyptus* genotype CEPT1864	S.F. Chen, G.Q. Li & Q.C. Wang	–	–	–	–	–	–	MW285278	MW285520	–	–
*C. hongkongensis*	AA--	CSF16112	20181021-1-(172)	soil under *Eucalyptus* genotype CEPT1864	S.F. Chen, G.Q. Li & Q.C. Wang	–	–	–	–	–	–	MW285279	MW285521	–	–
*C. hongkongensis*	AA--	CSF16113	20181021-1-(172)	soil under *Eucalyptus* genotype CEPT1864	S.F. Chen, G.Q. Li & Q.C. Wang	–	–	–	–	–	–	MW285280	MW285522	–	–
*C. hongkongensis*	AA--	CSF16115	20181021-1-(172)	soil under *Eucalyptus* genotype CEPT1864	S.F. Chen, G.Q. Li & Q.C. Wang	–	–	–	–	–	–	MW285282	MW285524	–	–
*C. hongkongensis*	AAAA	CSF16120 ^9^	20181021-1-(198)	soil under *Eucalyptus* genotype CEPT1865	S.F. Chen, G.Q. Li & Q.C. Wang	–	–	–	–	–	–	MW285287	MW285529	MW290581	MW290651
*C. hongkongensis*	AA--	CSF16125	20181021-1-(214)	soil under *Eucalyptus* genotype CEPT1865	S.F. Chen, G.Q. Li & Q.C. Wang	–	–	–	–	–	–	MW285292	MW285534	–	–
*C. hongkongensis*	AA--	CSF16126	20181021-1-(214)	soil under *Eucalyptus* genotype CEPT1865	S.F. Chen, G.Q. Li & Q.C. Wang	–	–	–	–	–	–	MW285293	MW285535	–	–
*C. hongkongensis*	AA--	CSF16127	20181021-1-(214)	soil under *Eucalyptus* genotype CEPT1865	S.F. Chen, G.Q. Li & Q.C. Wang	–	–	–	–	–	–	MW285294	MW285536	–	–
*C. hongkongensis*	AA--	CSF16128	20181021-1-(214)	soil under *Eucalyptus* genotype CEPT1865	S.F. Chen, G.Q. Li & Q.C. Wang	–	–	–	–	–	–	MW285295	MW285537	–	–
*C. hongkongensis*	AA--	CSF16129	20181021-1-(214)	soil under *Eucalyptus* genotype CEPT1865	S.F. Chen, G.Q. Li & Q.C. Wang	–	–	–	–	–	–	MW285296	MW285538	–	–
*C. hongkongensis*	AAAA	CSF16149 ^9^	20181021-1-(258)	soil under *Eucalyptus* genotype CEPT1866	S.F. Chen, G.Q. Li & Q.C. Wang	–	–	–	–	–	–	MW285316	MW285554	MW290595	MW290665
*C. hongkongensis*	AA--	CSF16150	20181021-1-(258)	soil under *Eucalyptus* genotype CEPT1866	S.F. Chen, G.Q. Li & Q.C. Wang	–	–	–	–	–	–	MW285317	MW285555	–	–
*C. hongkongensis*	AA--	CSF16151	20181021-1-(258)	soil under *Eucalyptus* genotype CEPT1866	S.F. Chen, G.Q. Li & Q.C. Wang	–	–	–	–	–	–	MW285318	MW285556	–	–
*C. hongkongensis*	AA--	CSF16152	20181021-1-(258)	soil under *Eucalyptus* genotype CEPT1866	S.F. Chen, G.Q. Li & Q.C. Wang	–	–	–	–	–	–	MW285319	MW285557	–	–
*C. hongkongensis*	AA--	CSF16153	20181021-1-(260)	soil under *Eucalyptus* genotype CEPT1866	S.F. Chen, G.Q. Li & Q.C. Wang	–	–	–	–	–	–	MW285320	MW285558	–	–
*C. hongkongensis*	AA--	CSF16154	20181021-1-(260)	soil under *Eucalyptus* genotype CEPT1866	S.F. Chen, G.Q. Li & Q.C. Wang	–	–	–	–	–	–	MW285321	MW285559	–	–
*C. hongkongensis*	AA--	CSF16155	20181021-1-(260)	soil under *Eucalyptus* genotype CEPT1866	S.F. Chen, G.Q. Li & Q.C. Wang	–	–	–	–	–	–	MW285322	MW285560	–	–
*C. hongkongensis*	AA--	CSF16156	20181021-1-(260)	soil under *Eucalyptus* genotype CEPT1866	S.F. Chen, G.Q. Li & Q.C. Wang	–	–	–	–	–	–	MW285323	MW285561	–	–
*C. hongkongensis*	AAAA	CSF16159 ^9^	20181021-1-(274)	soil under *Eucalyptus* genotype CEPT1867	S.F. Chen, G.Q. Li & Q.C. Wang	–	–	–	–	–	–	MW285324	MW285562	MW290596	MW290666
*C. hongkongensis*	AA--	CSF16160	20181021-1-(274)	soil under *Eucalyptus* genotype CEPT1867	S.F. Chen, G.Q. Li & Q.C. Wang	–	–	–	–	–	–	MW285325	MW285563	–	–
*C. hongkongensis*	AA--	CSF16161	20181021-1-(274)	soil under *Eucalyptus* genotype CEPT1867	S.F. Chen, G.Q. Li & Q.C. Wang	–	–	–	–	–	–	MW285326	MW285564	–	–
*C. hongkongensis*	AA--	CSF16162	20181021-1-(274)	soil under *Eucalyptus* genotype CEPT1867	S.F. Chen, G.Q. Li & Q.C. Wang	–	–	–	–	–	–	MW285327	MW285565	–	–
*C. hongkongensis*	AA--	CSF16166	20181021-1-(278)	soil under *Eucalyptus* genotype CEPT1867	S.F. Chen, G.Q. Li & Q.C. Wang	–	–	–	–	–	–	MW285331	MW285569	–	–
*C. hongkongensis*	AA--	CSF16167	20181021-1-(280)	soil under *Eucalyptus* genotype CEPT1867	S.F. Chen, G.Q. Li & Q.C. Wang	–	–	–	–	–	–	MW285332	MW285570	–	–
*C. hongkongensis*	AA--	CSF16169	20181021-1-(280)	soil under *Eucalyptus* genotype CEPT1867	S.F. Chen, G.Q. Li & Q.C. Wang	–	–	–	–	–	–	MW285333	MW285571	–	–
*C. hongkongensis*	AA--	CSF16170	20181021-1-(280)	soil under *Eucalyptus* genotype CEPT1867	S.F. Chen, G.Q. Li & Q.C. Wang	–	–	–	–	–	–	MW285334	MW285572	–	–
*C. hongkongensis*	AA--	CSF16171	20181021-1-(284)	soil under *Eucalyptus* genotype CEPT1867	S.F. Chen, G.Q. Li & Q.C. Wang	–	–	–	–	–	–	MW285335	MW285573	–	–
*C. hongkongensis*	AA--	CSF16172	20181021-1-(284)	soil under *Eucalyptus* genotype CEPT1867	S.F. Chen, G.Q. Li & Q.C. Wang	–	–	–	–	–	–	MW285336	MW285574	–	–
*C. hongkongensis*	AA--	CSF16173	20181021-1-(284)	soil under *Eucalyptus* genotype CEPT1867	S.F. Chen, G.Q. Li & Q.C. Wang	–	–	–	–	–	–	MW285337	MW285575	–	–
*C. hongkongensis*	AA--	CSF16174	20181021-1-(284)	soil under *Eucalyptus* genotype CEPT1867	S.F. Chen, G.Q. Li & Q.C. Wang	–	–	–	–	–	–	MW285338	MW285576	–	–
*C. hongkongensis*	AA--	CSF16175	20181021-1-(286)	soil under *Eucalyptus* genotype CEPT1867	S.F. Chen, G.Q. Li & Q.C. Wang	–	–	–	–	–	–	MW285339	MW285577	–	–
*C. hongkongensis*	AA--	CSF16176	20181021-1-(286)	soil under *Eucalyptus* genotype CEPT1867	S.F. Chen, G.Q. Li & Q.C. Wang	–	–	–	–	–	–	MW285340	MW285578	–	–
*C. hongkongensis*	AA--	CSF16177	20181021-1-(286)	soil under *Eucalyptus* genotype CEPT1867	S.F. Chen, G.Q. Li & Q.C. Wang	–	–	–	–	–	–	MW285341	MW285579	–	–
*C. hongkongensis*	AA--	CSF16178	20181021-1-(286)	soil under *Eucalyptus* genotype CEPT1867	S.F. Chen, G.Q. Li & Q.C. Wang	–	–	–	–	–	–	MW285342	MW285580	–	–
*C. hongkongensis*	AA--	CSF16179	20181021-1-(290)	soil under *Eucalyptus* genotype CEPT1867	S.F. Chen, G.Q. Li & Q.C. Wang	–	–	–	–	–	–	MW285343	MW285581	–	–
*C. hongkongensis*	AA--	CSF16180	20181021-1-(290)	soil under *Eucalyptus* genotype CEPT1867	S.F. Chen, G.Q. Li & Q.C. Wang	–	–	–	–	–	–	MW285344	MW285582	–	–
*C. hongkongensis*	AA--	CSF16181	20181021-1-(290)	soil under *Eucalyptus* genotype CEPT1867	S.F. Chen, G.Q. Li & Q.C. Wang	–	–	–	–	–	–	MW285345	MW285583	–	–
*C. hongkongensis*	AA--	CSF16182	20181021-1-(290)	soil under *Eucalyptus* genotype CEPT1867	S.F. Chen, G.Q. Li & Q.C. Wang	–	–	–	–	–	–	MW285346	MW285584	–	–
*C. hongkongensis*	AA--	CSF16183	20181021-1-(290)	soil under *Eucalyptus* genotype CEPT1867	S.F. Chen, G.Q. Li & Q.C. Wang	–	–	–	–	–	–	MW285347	MW285585	–	–
*C. hongkongensis*	AA-A	CSF16184	20181021-1-(302)	soil under *Eucalyptus* genotype CEPT1868	S.F. Chen, G.Q. Li & Q.C. Wang	–	–	–	–	–	–	MW285348	MW285586	No	MW290668
*C. hongkongensis*	AA--	CSF16186	20181021-1-(302)	soil under *Eucalyptus* genotype CEPT1868	S.F. Chen, G.Q. Li & Q.C. Wang	–	–	–	–	–	–	MW285350	MW285588	–	–
*C. hongkongensis*	AAAA	CSF16187 ^9^	20181021-1-(302)	soil under *Eucalyptus* genotype CEPT1868	S.F. Chen, G.Q. Li & Q.C. Wang	–	–	–	–	–	–	MW285351	MW285589	MW290599	MW290670
*C. hongkongensis*	AAAA	CSF16188	20181021-1-(304)	soil under *Eucalyptus* genotype CEPT1868	S.F. Chen, G.Q. Li & Q.C. Wang	–	–	–	–	–	–	MW285352	MW285590	MW290600	MW290671
*C. hongkongensis*	AA--	CSF16189	20181021-1-(304)	soil under *Eucalyptus* genotype CEPT1868	S.F. Chen, G.Q. Li & Q.C. Wang	–	–	–	–	–	–	MW285353	MW285591	–	–
*C. hongkongensis*	AA--	CSF16190	20181021-1-(304)	soil under *Eucalyptus* genotype CEPT1868	S.F. Chen, G.Q. Li & Q.C. Wang	–	–	–	–	–	–	MW285354	MW285592	–	–
*C. hongkongensis*	-A--	CSF16191	20181021-1-(304)	soil under *Eucalyptus* genotype CEPT1868	S.F. Chen, G.Q. Li & Q.C. Wang	–	–	–	–	–	–	No	MW285593	–	–
*C. hongkongensis*	AA--	CSF16192	20181021-1-(306)	soil under *Eucalyptus* genotype CEPT1868	S.F. Chen, G.Q. Li & Q.C. Wang	–	–	–	–	–	–	MW285355	MW285594	–	–
*C. hongkongensis*	AA--	CSF16193	20181021-1-(306)	soil under *Eucalyptus* genotype CEPT1868	S.F. Chen, G.Q. Li & Q.C. Wang	–	–	–	–	–	–	MW285356	MW285595	–	–
*C. hongkongensis*	AA--	CSF16194	20181021-1-(306)	soil under *Eucalyptus* genotype CEPT1868	S.F. Chen, G.Q. Li & Q.C. Wang	–	–	–	–	–	–	MW285357	MW285596	–	–
*C. hongkongensis*	AA--	CSF16195	20181021-1-(306)	soil under *Eucalyptus* genotype CEPT1868	S.F. Chen, G.Q. Li & Q.C. Wang	–	–	–	–	–	–	MW285358	MW285597	–	–
*C. hongkongensis*	-A--	CSF16196	20181021-1-(306)	soil under *Eucalyptus* genotype CEPT1868	S.F. Chen, G.Q. Li & Q.C. Wang	–	–	–	–	–	–	No	MW285598	–	–
*C. hongkongensis*	AA--	CSF16197	20181021-1-(306)	soil under *Eucalyptus* genotype CEPT1868	S.F. Chen, G.Q. Li & Q.C. Wang	–	–	–	–	–	–	MW285359	MW285599	–	–
*C. hongkongensis*	-A--	CSF16199	20181021-1-(308)	soil under *Eucalyptus* genotype CEPT1868	S.F. Chen, G.Q. Li & Q.C. Wang	–	–	–	–	–	–	No	MW285601	–	–
*C. hongkongensis*	AA--	CSF16202	20181021-1-(314)	soil under *Eucalyptus* genotype CEPT1868	S.F. Chen, G.Q. Li & Q.C. Wang	–	–	–	–	–	–	MW285363	MW285604	–	–
*C. hongkongensis*	AA--	CSF16203	20181021-1-(314)	soil under *Eucalyptus* genotype CEPT1868	S.F. Chen, G.Q. Li & Q.C. Wang	–	–	–	–	–	–	MW285364	MW285605	–	–
*C. hongkongensis*	AA--	CSF16204	20181021-1-(314)	soil under *Eucalyptus* genotype CEPT1868	S.F. Chen, G.Q. Li & Q.C. Wang	–	–	–	–	–	–	MW285365	MW285606	–	–
*C. hongkongensis*	AA--	CSF16205	20181021-1-(314)	soil under *Eucalyptus* genotype CEPT1868	S.F. Chen, G.Q. Li & Q.C. Wang	–	–	–	–	–	–	MW285366	MW285607	–	–
*C. hongkongensis*	AA--	CSF16207	20181021-1-(320)	soil under *Eucalyptus* genotype CEPT1868	S.F. Chen, G.Q. Li & Q.C. Wang	–	–	–	–	–	–	MW285367	MW285608	–	–
*C. hongkongensis*	AA--	CSF16208	20181021-1-(320)	soil under *Eucalyptus* genotype CEPT1868	S.F. Chen, G.Q. Li & Q.C. Wang	–	–	–	–	–	–	MW285368	MW285609	–	–
*C. hongkongensis*	AA--	CSF16210	20181021-1-(320)	soil under *Eucalyptus* genotype CEPT1868	S.F. Chen, G.Q. Li & Q.C. Wang	–	–	–	–	–	–	MW285370	MW285611	–	–
*C. hongkongensis*	AA--	CSF16212	20181021-1-(322)	soil under *Eucalyptus* genotype CEPT1868	S.F. Chen, G.Q. Li & Q.C. Wang	–	–	–	–	–	–	MW285372	MW285613	–	–
*C. hongkongensis*	AA--	CSF16213	20181021-1-(322)	soil under *Eucalyptus* genotype CEPT1868	S.F. Chen, G.Q. Li & Q.C. Wang	–	–	–	–	–	–	MW285373	MW285614	–	–
*C. hongkongensis*	AA--	CSF16214	20181021-1-(322)	soil under *Eucalyptus* genotype CEPT1868	S.F. Chen, G.Q. Li & Q.C. Wang	–	–	–	–	–	–	MW285374	MW285615	–	–
*C. hongkongensis*	AA--	CSF16215	20181021-1-(322)	soil under *Eucalyptus* genotype CEPT1868	S.F. Chen, G.Q. Li & Q.C. Wang	–	–	–	–	–	–	MW285375	MW285616	–	–
*C. hongkongensis*	AA--	CSF16218	20181021-1-(324)	soil under *Eucalyptus* genotype CEPT1868	S.F. Chen, G.Q. Li & Q.C. Wang	–	–	–	–	–	–	MW285378	MW285619	–	–
*C. hongkongensis*	AA--	CSF16219	20181021-1-(324)	soil under *Eucalyptus* genotype CEPT1868	S.F. Chen, G.Q. Li & Q.C. Wang	–	–	–	–	–	–	MW285379	MW285620	–	–
*C. hongkongensis*	AA--	CSF16221	20181021-1-(326)	soil under *Eucalyptus* genotype CEPT1868	S.F. Chen, G.Q. Li & Q.C. Wang	–	–	–	–	–	–	MW285380	MW285621	–	–
*C. hongkongensis*	AA--	CSF16222	20181021-1-(326)	soil under *Eucalyptus* genotype CEPT1868	S.F. Chen, G.Q. Li & Q.C. Wang	–	–	–	–	–	–	MW285381	MW285622	–	–
*C. hongkongensis*	AA--	CSF16223	20181021-1-(330)	soil under *Eucalyptus* genotype CEPT1868	S.F. Chen, G.Q. Li & Q.C. Wang	–	–	–	–	–	–	MW285382	MW285623	–	–
*C. hongkongensis*	AA--	CSF16224	20181021-1-(330)	soil under *Eucalyptus* genotype CEPT1868	S.F. Chen, G.Q. Li & Q.C. Wang	–	–	–	–	–	–	MW285383	MW285624	–	–
*C. hongkongensis*	AA--	CSF16225	20181021-1-(330)	soil under *Eucalyptus* genotype CEPT1868	S.F. Chen, G.Q. Li & Q.C. Wang	–	–	–	–	–	–	MW285384	MW285625	–	–
*C. hongkongensis*	AA--	CSF16226	20181021-1-(330)	soil under *Eucalyptus* genotype CEPT1868	S.F. Chen, G.Q. Li & Q.C. Wang	–	–	–	–	–	–	MW285385	MW285626	–	–
*C. hongkongensis*	A---	CSF16231	20181021-1-(336)	soil under *Eucalyptus* genotype CEPT1868	S.F. Chen, G.Q. Li & Q.C. Wang	–	–	–	–	–	–	MW285390	No	–	–
*C. hongkongensis*	AA--	CSF16234	20181021-1-(336)	soil under *Eucalyptus* genotype CEPT1868	S.F. Chen, G.Q. Li & Q.C. Wang	–	–	–	–	–	–	MW285391	MW285631	–	–
*C. hongkongensis*	AAAA	CSF16237 ^6,9,10,11^	20181021-1-(350)	soil under *Eucalyptus* genotype CEPT1869	S.F. Chen, G.Q. Li & Q.C. Wang	MAT1-1-1_GT1	MAT1-2-1_GT2	HO	homothallic	MW290800	MW319763	MW285394	MW285634	MW290610	MW290682
*C. hongkongensis*	AA--	CSF16238	20181021-1-(350)	soil under *Eucalyptus* genotype CEPT1869	S.F. Chen, G.Q. Li & Q.C. Wang	–	–	–	–	–	–	MW285395	MW285635	–	–
*C. hongkongensis*	AA--	CSF16239	20181021-1-(350)	soil under *Eucalyptus* genotype CEPT1869	S.F. Chen, G.Q. Li & Q.C. Wang	–	–	–	–	–	–	MW285396	MW285636	–	–
*C. hongkongensis*	AA--	CSF16240	20181021-1-(350)	soil under *Eucalyptus* genotype CEPT1869	S.F. Chen, G.Q. Li & Q.C. Wang	–	–	–	–	–	–	MW285397	MW285637	–	–
*C. hongkongensis*	ABAA	CSF16096 ^6,9^	20181021-1-(142)	soil under *Eucalyptus* genotype CEPT1864	S.F. Chen, G.Q. Li & Q.C. Wang	MAT1-1-1_GT1	MAT1-2-1_GT2	HO	homothallic	MW290764	MW319747	MW285264	MW285506	MW290572	MW290642
*C. hongkongensis*	ABAA	CSF16097	20181021-1-(142)	soil under *Eucalyptus* genotype CEPT1864	S.F. Chen, G.Q. Li & Q.C. Wang	–	–	–	–	–	–	MW285265	MW285507	MW290573	MW290643
*C. hongkongensis*	ABAA	CSF16098	20181021-1-(142)	soil under *Eucalyptus* genotype CEPT1864	S.F. Chen, G.Q. Li & Q.C. Wang	–	–	–	–	–	–	MW285266	MW285508	MW290574	MW290644
*C. hongkongensis*	ABAA	CSF16099	20181021-1-(142)	soil under *Eucalyptus* genotype CEPT1864	S.F. Chen, G.Q. Li & Q.C. Wang	–	–	–	–	–	–	MW285267	MW285509	MW290575	MW290645
*C. hongkongensis*	ABAA	CSF16100	20181021-1-(142)	soil under *Eucalyptus* genotype CEPT1864	S.F. Chen, G.Q. Li & Q.C. Wang	–	–	–	–	–	–	MW285268	MW285510	MW290576	MW290646
*C. hongkongensis*	ABAA	CSF16216 ^6,9^	20181021-1-(324)	soil under *Eucalyptus* genotype CEPT1868	S.F. Chen, G.Q. Li & Q.C. Wang	MAT1-1-1_GT2	MAT1-2-1_GT2	HO	homothallic	MW290795	MW319760	MW285376	MW285617	MW290604	MW290675
*C. hongkongensis*	AB-A	CSF16217	20181021-1-(324)	soil under *Eucalyptus* genotype CEPT1868	S.F. Chen, G.Q. Li & Q.C. Wang	–	–	–	–	–	–	MW285377	MW285618	No	MW290676
*C. hongkongensis*	ACAA	CSF16121 ^6,9,10^	20181021-1-(206)	soil under *Eucalyptus* genotype CEPT1865	S.F. Chen, G.Q. Li & Q.C. Wang	MAT1-1-1_GT2	MAT1-2-1_GT2	HO	homothallic	MW290771	MW319751	MW285288	MW285530	MW290582	MW290652
*C. hongkongensis*	ACAA	CSF16122	20181021-1-(206)	soil under *Eucalyptus* genotype CEPT1865	S.F. Chen, G.Q. Li & Q.C. Wang	–	–	–	–	–	–	MW285289	MW285531	MW290583	MW290653
*C. hongkongensis*	ACAA	CSF16123	20181021-1-(206)	soil under *Eucalyptus* genotype CEPT1865	S.F. Chen, G.Q. Li & Q.C. Wang	–	–	–	–	–	–	MW285290	MW285532	MW290584	MW290654
*C. hongkongensis*	ACAA	CSF16124 ^6^	20181021-1-(206)	soil under *Eucalyptus* genotype CEPT1865	S.F. Chen, G.Q. Li & Q.C. Wang	MAT1-1-1_GT2	MAT1-2-1_GT2	HO	homothallic	MW290772	MW319752	MW285291	MW285533	MW290585	MW290655
*C. hongkongensis*	ADA-	CSF16198	20181021-1-(308)	soil under *Eucalyptus* genotype CEPT1868	S.F. Chen, G.Q. Li & Q.C. Wang	–	–	–	–	–	–	MW285360	MW285600	MW290601	No
*C. hongkongensis*	AD--	CSF16200	20181021-1-(308)	soil under *Eucalyptus* genotype CEPT1868	S.F. Chen, G.Q. Li & Q.C. Wang	–	–	–	–	–	–	MW285361	MW285602	No	No
*C. hongkongensis*	ADAA	CSF16201	20181021-1-(308)	soil under *Eucalyptus* genotype CEPT1868	S.F. Chen, G.Q. Li & Q.C. Wang	–	–	–	–	–	–	MW285362	MW285603	MW290602	MW290672
*C. hongkongensis*	ADAA	CSF16227 ^6^	20181021-1-(334)	soil under *Eucalyptus* genotype CEPT1868	S.F. Chen, G.Q. Li & Q.C. Wang	MAT1-1-1_GT1	MAT1-2-1_GT2	HO	homothallic	MW290796	MW319761	MW285386	MW285627	MW290605	MW290677
*C. hongkongensis*	ADAA	CSF16228	20181021-1-(334)	soil under *Eucalyptus* genotype CEPT1868	S.F. Chen, G.Q. Li & Q.C. Wang	–	–	–	–	–	–	MW285387	MW285628	MW290606	MW290678
*C. hongkongensis*	ADAA	CSF16229	20181021-1-(334)	soil under *Eucalyptus* genotype CEPT1868	S.F. Chen, G.Q. Li & Q.C. Wang	–	–	–	–	–	–	MW285388	MW285629	MW290607	MW290679
*C. hongkongensis*	ADAA	CSF16230 ^6,9,10,11^	20181021-1-(334)	soil under *Eucalyptus* genotype CEPT1868	S.F. Chen, G.Q. Li & Q.C. Wang	MAT1-1-1_GT1	MAT1-2-1_GT2	HO	homothallic	MW290797	MW319762	MW285389	MW285630	MW290608	MW290680
*C. hongkongensis*	BAAA	CSF16091 ^6^	20181021-1-(86)	soil under *Eucalyptus* genotype CEPT1862	S.F. Chen, G.Q. Li & Q.C. Wang	MAT1-1-1_GT1	MAT1-2-1_GT2	HO	homothallic	MW290762	MW319743	MW285259	MW285501	MW290569	MW290639
*C. hongkongensis*	BAAA	CSF16092	20181021-1-(86)	soil under *Eucalyptus* genotype CEPT1862	S.F. Chen, G.Q. Li & Q.C. Wang	–	–	–	–	–	–	MW285260	MW285502	MW290570	MW290640
*C. hongkongensis*	BAAA	CSF16093 ^6,9^	20181021-1-(86)	soil under *Eucalyptus* genotype CEPT1862	S.F. Chen, G.Q. Li & Q.C. Wang	MAT1-1-1_GT1	MAT1-2-1_GT2	HO	homothallic	MW290763	MW319744	MW285261	MW285503	MW290571	MW290641
*C. hongkongensis*	CAAA	CSF16145 ^6,9,10,11^	20181021-1-(256)	soil under *Eucalyptus* genotype CEPT1866	S.F. Chen, G.Q. Li & Q.C. Wang	MAT1-1-1_GT1	MAT1-2-1_GT2	HO	homothallic	MW290788	MW319757	MW285312	MW285550	MW290591	MW290661
*C. hongkongensis*	CAAA	CSF16146	20181021-1-(256)	soil under *Eucalyptus* genotype CEPT1866	S.F. Chen, G.Q. Li & Q.C. Wang	–	–	–	–	–	–	MW285313	MW285551	MW290592	MW290662
*C. hongkongensis*	CAAA	CSF16147	20181021-1-(256)	soil under *Eucalyptus* genotype CEPT1866	S.F. Chen, G.Q. Li & Q.C. Wang	–	–	–	–	–	–	MW285314	MW285552	MW290593	MW290663
*C. hongkongensis*	CAAA	CSF16148 ^6^	20181021-1-(256)	soil under *Eucalyptus* genotype CEPT1866	S.F. Chen, G.Q. Li & Q.C. Wang	MAT1-1-1_GT1	MAT1-2-1_GT2	HO	homothallic	MW290789	MW319758	MW285315	MW285553	MW290594	MW290664
*C. aconidialis*	A-AA	CSF16130 ^6,9,10,11^	20181021-1-(216)	soil under *Eucalyptus* genotype CEPT1865	S.F. Chen, G.Q. Li & Q.C. Wang	MAT1-1-1_GT1	MAT1-2-1-GT1	HO	homothallic	MW290773	MW319753	MW285297	No	MW290586	MW290656
*C. aconidialis*	A-AA	CSF16131 ^6,9,10^	20181021-1-(216)	soil under *Eucalyptus* genotype CEPT1865	S.F. Chen, G.Q. Li & Q.C. Wang	MAT1-1-1_GT1	MAT1-2-1-GT1	HO	homothallic	MW290774	MW319754	MW285298	No	MW290587	MW290657
*C. aconidialis*	A-AA	CSF16132 ^6,9^	20181021-1-(216)	soil under *Eucalyptus* genotype CEPT1865	S.F. Chen, G.Q. Li & Q.C. Wang	MAT1-1-1_GT1	MAT1-2-1-GT1	HO	homothallic	MW290775	MW319755	MW285299	No	MW290588	MW290658
*C. aconidialis*	A-AA	CSF16133 ^6,9,10,11^	20181021-1-(216)	soil under *Eucalyptus* genotype CEPT1865	S.F. Chen, G.Q. Li & Q.C. Wang	MAT1-1-1_GT1	MAT1-2-1-GT1	HO	homothallic	MW290776	MW319756	MW285300	No	MW290589	MW290659

^1^ Genotype within each *Calonectria* species, determined by sequences of the *tef1*, *tub2*, *cmdA* and *his3* regions; “-” means not available. ^2^ CSF: Culture Collection located at China Eucalypt Research Centre (CERC), Chinese Academy of Forestry, ZhanJiang, GuangDong Province, China. ^3^ Genotype of each mating gene within each *Calonectria* species, determined by sequences of *Mat1-1-1* or *Mat1-2-1*; GT1, GT2 mean genotype 1 and genotype 2, respectively. ^4^ HE = Heterothallic; HO = Homothallic; P_HE = Putative heterothallic. ^5^ *tef1* = translation elongation factor 1-alpha; *tub2* = β-tubulin; *cmdA* = calmodulin; *his3* = histone H3. ^6^ Isolates used for mating studies. ^7^ “No” represents the relative locus was not successfully amplified in the current study. ^8^ “–” represents the relative locus was not amplified in the current study. ^9^ Isolates used for phylogenetic analyses. ^10^ Isolates used for morphological studies. ^11^ Isolates used for pathogenicity tests.

**Table A2:** Isolates from other studies and used in the phylogenetic analyses for this study.

Species Code ^1^	Species	Isolate No. ^2,3^	Other Collection Number ^3^	Hosts	Area of Occurrence	Collector	GenBank Accession Numbers ^4^				References or Source of Data
							*tef1*	*tub2*	*cmdA*	*his3*	
B1	*Calonectria acaciicola*	CMW 47173^T^	CBS 143557	Soil (*Acacia auriculiformis* plantation)	Do Luong, Nghe An, Vietnam	N.Q. Pham and T.Q. Pham	MT412690	MT412930	MT335160	MT335399	[28,36]
		CMW 47174	CBS 143558	Soil (*A. auriculiformis* plantation)	Do Luong, Nghe An, Vietnam	N.Q. Pham and T.Q. Pham	MT412691	MT412931	MT335161	MT335400	[28,36]
B2	*C. acicola*	CMW 30996^T^	–	*Phoenix canariensis*	Northland, New Zealand	H. Pearson	MT412692	MT412932	MT335162	MT335401	[28,49,50]
		CBS 114812	CMW 51216	*P. canariensis*	Northland, New Zealand	H. Pearson	MT412693	MT412933	MT335163	MT335402	[28,49,50]
B4	*C. aconidialis*	CMW 35174^T^	CBS 136086; CERC 1850	Soil (*Eucalyptus* plantation)	HaiNan, China	X. Mou and S.F. Chen	MT412695	N/A ^5^	MT335165	MT335404	[28,29]
		CMW 35384	CBS 136091; CERC 1886	Soil (*Eucalyptus* plantation)	HaiNan, China	X. Mou and S.F. Chen	MT412696	N/A	MT335166	MT335405	[28,29]
B5	*C. aeknauliensis*	CMW 48253^T^	CBS 143559	Soil (*Eucalyptus* plantation)	Aek Nauli, North Sumatra, Indonesia	M.J. Wingfield	MT412710	N/A	MT335180	MT335419	[28,36]
		CMW 48254	CBS 143560	Soil (*Eucalyptus* plantation)	Aek Nauli, North Sumatra, Indonesia	M.J. Wingfield	MT412711	N/A	MT335181	MT335420	[28,36]
B8	*C. asiatica*	CBS 114073^T^	CMW 23782; CPC 3900	Debris (leaf litter)	Prathet Thai, Thailand	N.L. Hywel-Jones	AY725705	AY725616	AY725741	AY725658	[43,50]
B9	*C. auriculiformis*	CMW 47178^T^	CBS 143561	Soil (*A. auriculiformis* plantation)	Hau Loc, Thanh Hoa, Vietnam	N.Q. Pham and T.Q. Pham	MT412721	MT412944	MT335190	MT335430	[28,36]
		CMW 47179	CBS 143562	Soil (*A. auriculiformis* plantation)	Hau Loc, Thanh Hoa, Vietnam	N.Q. Pham and T.Q. Pham	MT412722	MT412945	MT335191	MT335431	[28,36]
B10	*C. australiensis*	CMW 23669^T^	CBS 112954; CPC 4714	*Ficus pleurocarpa*	Queensland, Australia	C. Pearce and B. Paulus	MT412723	MT412946	MT335192	MT335432	[28,50,51]
B14	*C. brasiliensis*	CBS 230.51^T^	IMI 299576	*Eucalyptus* sp.	Ceara state, Brazil	T.R. Ciferri	MT412731	MT412953	MT335200	MT335440	[23,28,52]
		CMW 32949	CBS 114257; CPC 1944	*Eucalyptus* sp.	Aracruz, Brazil	A.C. Alfenas	MT412732	MT412954	MT335201	MT335441	[28,50]
B17	*C. brassicicola*	CBS 112841^T^	CMW 51206; CPC 4552	Soil (*Brassica* sp.)	Indonesia	M.J. Wingfield	KX784689	KX784619	KX784561	N/A	[32]
B19	*C. bumicola*	CMW 48257^T^	CBS 143575	Soil (*Eucalyptus* plantation)	Aek Nauli, North Sumatra, Indonesia	M.J. Wingfield	MT412736	N/A	MT335205	MT335445	[28,36]
B20	*C. canadiana*	CMW 23673^T^	CBS 110817; STE-U 499	*Picea* sp.	Canada	S. Greifenhagen	MT412737	MT412958	MT335206	MT335446	[23,28,53,54]
		CERC 8952	–	Soil	HeNan, China	S.F. Chen	MT412821	MT413035	MT335290	MT335530	[28,33]
B22	*C. cerciana*	CMW 25309^T^	CBS 123693	*E. urophylla × E. grandis* hybrid cutting	CERC nursery, GuangDong, China	M.J. Wingfield and X.D. Zhou	MT412742	MT412963	MT335211	MT335451	[28,30]
		CMW 25290	CBS 123695	*E. urophylla × E. grandis* hybrid cutting	CERC nursery, GuangDong, China	M.J. Wingfield and X.D. Zhou	MT412743	MT412964	MT335212	MT335452	[28,30]
B23	*C. chinensis*	CMW 23674^T^	CBS 114827; CPC 4101	Soil	Hong Kong, China	E.C.Y. Liew	MT412751	MT412972	MT335220	MT335460	[28,43,50]
		CMW 30986	CBS 112744; CPC 4104	Soil	Hong Kong, China	E.C.Y. Liew	MT412752	MT412973	MT335221	MT335461	[28,43,50]
B26	*C. cochinchinensis*	CMW 49915^T^	CBS 143567	Soil (*Hevea brasiliensis* plantation)	Duong Minh Chau, Tay Ninh, Vietnam	N.Q. Pham, Q.N. Dang and T.Q. Pham	MT412756	MT412977	MT335225	MT335465	[28,36]
		CMW 47186	CBS 143568	Soil (*A. auriculiformis* plantation)	Song May, Dong Nai, Vietnam	N.Q. Pham and T.Q. Pham	MT412757	MT412978	MT335226	MT335466	[28,36]
B29	*C. colombiensis*	CMW 23676^T^	CBS 112220; CPC 723	Soil (*E. grandis* trees)	La Selva, Colombia	M.J. Wingfield	MT412759	MT412980	MT335228	MT335468	[28,43]
		CMW 30985	CBS 112221; CPC 724	Soil (*E. grandis* trees)	La Selva, Colombia	M.J. Wingfield	MT412760	MT412981	MT335229	MT335469	[28,43]
B30	*C. crousiana*	CMW 27249^T^	CBS 127198	*E. grandis*	FuJian, China	M.J. Wingfield	MT412761	MT412982	MT335230	MT335470	[18,28]
		CMW 27253	CBS 127199	*E. grandis*	FuJian, China	M.J. Wingfield	MT412762	MT412983	MT335231	MT335471	[18,28]
B31	*C. curvispora*	CMW 23693^T^	CBS 116159; CPC 765	Soil	Tamatave, Madagascar	P.W. Crous	MT412763	N/A	MT335232	MT335472	[23,28,29,50,55]
		CMW 48245	CBS 143565	Soil (*Eucalyptus* plantation)	Aek Nauli, North Sumatra, Indonesia	M.J. Wingfield	MT412764	N/A	MT335233	MT335473	[28,36]
B32	*C. cylindrospora*	CBS 119670	CMW 51310; CPC 12766	*Pistacia lentiscus*	Italy	N/A	MT412767	MT412985	MT335236	MT335476	[28,29,32,56]
		CMW 30978	CBS 110666; P90.1479; STE-U 497	*Ilex vomitoria*	Florida, USA	N.E. El-Gholl	MT412768	MT412986	MT335237	MT335477	[23,28,50,56]
B44	*C. hawksworthii*	CBS 111870^T^	CMW 51194; CPC 2405	*Nelumbo nucifera*	Pamplemousses garden, Mauritius	A. Peerally	MT412785	MT413003	MT335254	MT335494	[23,28]
		CMW 31393	CBS 136641	*E. urophylla* × *E. grandis*	GuangXi, China	X. Zhou and G. Zhao	MT412778	MT412996	MT335247	MT335487	[28,29]
B46	*C. heveicola*	CMW 49913^T^	CBS 143570	Soil (*Hevea brasiliensis* plantation)	Bau Bang, Binh Duong, Vietnam	N.Q. Pham, Q.N. Dang and T.Q. Pham	MT412786	MT413004	MT335255	MT335495	[28,36]
		CMW 49928	CBS 143571	Soil	Bu Gia Map National Park, Binh Phuoc, Vietnam	N.Q. Pham, Q.N. Dang and T.Q. Pham	MT412811	MT413025	MT335280	MT335520	[28,36]
B48	*C. hongkongensis*	CBS 114828^T^	CMW 51217; CPC 4670	Soil	Hong Kong, China	M.J. Wingfield	MT412789	MT413007	MT335258	MT335498	[28,43]
		CERC 3570	CMW 47271	Soil (*Eucalyptus* plantation)	BeiHai, GuangXi, China	S.F. Chen, J.Q. Li and G.Q. Li	MT412791	MT413009	MT335260	MT335500	[19,28]
B51	*C. ilicicola*	CMW 30998^T^	CBS 190.50; IMI 299389; STE-U 2482	*Solanum tuberosum*	Bogor, Java, Indonesia	K.B. Boedijn and J. Reitsma	MT412797	N/A	MT335266	MT335506	[23,28,50]
B52	*C. indonesiae*	CMW 23683^T^	CBS 112823; CPC 4508	*Syzygium aromaticum*	Warambunga, Indonesia	M.J. Wingfield	MT412798	MT413015	MT335267	MT335507	[28,43]
		CBS 112840	CMW 51205; CPC 4554	*S. aromaticum*	Warambunga, Indonesia	M.J. Wingfield	MT412799	MT413016	MT335268	MT335508	[28,43]
B54	*C. insularis*	CMW 30991^T^	CBS 114558; CPC 768	Soil	Tamatave, Madagascar	P.W. Crous	MT412800	MT413017	MT335269	MT335509	[28,32,50,57]
		CMW 30992	CBS 114559; CPC 954	Soil	Conejos, Veracruz, Mexico	M.J. Wingfield	MT412801	MT413018	MT335270	MT335510	[28,32,50]
B55	*C. kyotensis*	CBS 114525^T^	ATCC 18834; CMW 51824; CPC 2367	*Robinia pseudoacacia*	Japan	T. Terashita	MT412802	MT413019	MT335271	MT335511	[23,28,32]
		CBS 114550	CMW 51825; CPC 2351	Soil	China	M.J. Wingfield	MT412777	MT412995	MT335246	MT335486	[28,32]
B56	*C. lageniformis*	CBS 111324^T^	CMW 51177; CPC 1473	Leaf of *Eucalyptus* sp.	Rivière Noire, Mauritius	H. Smith	KX784702	KX784632	KX784574	N/A	[32,58]
B57	*C. lantauensis*	CERC 3302^T^	CBS 142888; CMW 47252	Soil	LiDao, Hong Kong, China	M.J. Wingfield and S.F. Chen	MT412803	N/A	MT335272	MT335512	[19,28]
		CERC 3301	CBS 142887; CMW 47251	Soil	LiDao, Hong Kong, China	M.J. Wingfield and S.F. Chen	MT412804	N/A	MT335273	MT335513	[19,28]
B58	*C. lateralis*	CMW 31412^T^	CBS 136629	Soil (*Eucalyptus* plantation)	GuangXi, China	X. Zhou, G. Zhao and F. Han	MT412805	MT413020	MT335274	MT335514	[28,29]
B63	*C. lombardiana*	CMW 30602^T^	CBS 112634; CPC 4233; Lynfield 417	*Xanthorrhoea australis*	Victoria, Australia	T. Baigent	MT412926	MT413133	MT335395	MT335635	[23,30,51]
B66	*C. malesiana*	CMW 23687^T^	CBS 112752; CPC 4223	Soil	Northern Sumatra, Indonesia	M.J. Wingfield	MT412817	MT413031	MT335286	MT335526	[28,43]
		CBS 112710	CMW 51199; CPC 3899	Leaf litter	Prathet, Thailand	N.L. Hywel-Jones	MT412818	MT413032	MT335287	MT335527	[28,43]
B67	*C. maranhensis*	CBS 134811^T^	LPF142	*Eucalyptus* sp. (leaf)	Açailandia, Maranhao, Brazil	A.C. Alfenas	KM395861	KM395948	KM396035	KM396118	[26]
		CBS 134812	LPF143	*Eucalyptus* sp. (leaf)	Açailandia, Maranhao, Brazil	A.C. Alfenas	KM395862	KM395949	KM396036	KM396119	[26]
B74	*C. multiseptata*	CMW 23692^T^	CBS 112682; CPC 1589	*E. grandis*	North Sumatra, Indonesia	M.J. Wingfield	MT412830	MT413044	MT335299	MT335539	[23,28,51,59]
B80	*C. pacifica*	CMW 16726^T^	A1568; CBS 109063;IMI 354528;STE-U 2534	*Araucaria heterophylla*	Hawaii, USA	M. Aragaki	MT412842	N/A	MT335311	MT335551	[23,28,43,53]
		CMW 30988	CBS 114038	*Ipomoea aquatica*	Auckland, New Zealand	C.F. Hill	MT412843	N/A	MT335312	MT335552	[23,28,43,50]
B86	*C. penicilloides*	CMW 23696^T^	CBS 174. 55; STE-U 2388	*Prunus* sp.	Hatizyo Island, Japan	M. Ookubu	MT412869	MT413081	MT335338	MT335578	[23,28]
B89	*C. plurilateralis*	CBS 111401^T^	CMW 51178; CPC 1637	Soil	Ecuador	M.J. Wingfield	MT412871	MT413083	MT335340	MT335580	[28,32]
B90	*C. propaginicola*	CBS 134815^T^	LPF220	*Eucalyptus* sp. (seedling)	Santana, Pará, Brazil	A.C. Alfenas	KM395866	KM395953	KM396040	KM396123	[26]
		CBS 134816	LPF222	*Eucalyptus* sp. (seedling)	Santana, Pará, Brazil	A.C. Alfenas	KM395867	KM395954	KM396041	KM396124	[26]
B97	*C. pseudoreteaudii*	CMW 25310^T^	CBS 123694	*E. urophylla* × *E. grandis*	GuangDong, China	M.J. Wingfield and X.D. Zhou	MT412885	MT413096	MT335354	MT335594	[28,30]
		CMW 25292	CBS 123696	*E. urophylla* × *E. grandis*	GuangDong, China	M.J. Wingfield and X.D. Zhou	MT412886	MT413097	MT335355	MT335595	[28,30]
B104	*C. queenslandica*	CMW 30604^T^	CBS 112146; CPC 3213	*E. urophylla*	Lannercost, Queensland, Australia	B. Brown	MT412898	MT413108	MT335367	MT335607	[28,30,44]
		CMW 30603	CBS 112155; CPC 3210	*E. pellita*	Lannercost, Queensland, Australia	P.Q Thu and K.M. Old	MT412899	MT413109	MT335368	MT335608	[28,30,44]
B106	*C. reteaudii*	CMW 30984^T^	CBS 112144; CPC 3201	*E. camaldulensis*	Chon Thanh, Binh Phuoc, Vietnam	M.J. Dudzinski and P.Q. Thu	MT412901	MT413111	MT335370	MT335610	[23,28,44,51]
		CMW 16738	CBS 112143; CPC 3200	*Eucalyptus* leaves	Binh Phuoc, Vietnam	M.J. Dudzinski and P.Q. Thu	MT412902	MT413112	MT335371	MT335611	[23,28,44,51]
B112	*C. sumatrensis*	CMW 23698^T^	CBS 112829; CPC 4518	Soil	Northern Sumatra, Indonesia	M.J. Wingfield	MT412913	N/A	MT335382	MT335622	[28,43]
		CMW 30987	CBS 112934; CPC 4516	Soil	Northern Sumatra, Indonesia	M.J. Wingfield	MT412914	N/A	MT335383	MT335623	[28,43]
B113	*C. syzygiicola*	CBS 112831^T^	CMW 51204; CPC 4511	*Syzygium aromaticum*	Sumatra, Indonesia	M.J. Wingfield	KX784736	KX784663	N/A	N/A	[32]
B115	*C. tonkinensis*	CMW 47430^T^	CBS 143576	Soil (*Eucalyptus* plantation)	Bavi, Hanoi, Vietnam	N.Q. Pham and T.Q. Pham	MT412915	MT413122	MT335384	MT335624	[28,36]
B116	*C. uniseptata*	CBS 413.67^T^	CMW 23678; CPC 2391; IMI 299577	*Paphiopedilum callosum*	Celle, Germany	W. Gerlach	GQ267307	GQ267208	GQ267379	GQ267248	[32]
B118	*C. variabilis*	CMW 3187^T^	AR2675; CBS 114677; CPC 2436	*Schefflera morototoni*	Pará, Brazil	F.C. de Albuquerque	MT412923	MT413130	MT335392	MT335632	[23,28,32,50,60]
		CMW 2914	CBS 112691; CPC 2506	*Theobroma grandiflorum*	Pará, Brazil	F. Carneiro	MT412924	MT413131	MT335393	MT335633	[23,28,32,50,60]
B120	*C. yunnanensis*	CERC 5339^T^	CBS 142897; CMW 47644	Soil (*Eucalyptus* plantation)	YunNan, China	S.F. Chen and J.Q. Li	MT412927	MT413134	MT335396	MT335636	[19,28]
		CERC 5337	CBS 142895; CMW 47642	Soil (*Eucalyptus* plantation)	YunNan, China	S.F. Chen and J.Q. Li	MT412928	MT413135	MT335397	MT335637	[19,28]
	*Curvicladiella cignea*	CBS 109167^T^	CPC 1595; MUCL 40269	Decaying leaf	French Guiana	C. Decock	KM231867	KM232002	KM231287	KM231461	[51,56,61]
		CBS 109168	CPC 1594; MUCL 40268	Decaying seed	French Guiana	C. Decock	KM231868	KM232003	KM231286	KM231460	[51,56,61]

^1^ Codes (B1 to B120) of the 120 accepted *Calonectria* species resulting from Liu and co-authors [28]. ^2^ T: ex-type isolates of the species. ^3^ AR: Amy Y. Rossman working collection; ATCC: American Type Culture Collection, Virginia, USA; CBS: Westerdijk Fungal Biodiversity Institute, Utrecht, The Netherlands; CERC: China Eucalypt Research Centre, ZhanJiang, GuangDong Province, China; CMW: Culture collection of the Forestry and Agricultural Biotechnology Institute (FABI), University of Pretoria, Pretoria, South Africa; CPC: Pedro Crous working collection housed at Westerdijk Fungal Biodiversity Institute; IMI: International Mycological Institute, CABI Bioscience, Egham, Bakeham Lane, UK; MUCL: Mycotheque, Laboratoire de Mycologie Systematique st Appliqee, I’Universite, Louvian-la-Neuve, Belgium; STE-U: Department of Plant Pathology, University of Stellenbosch, South Africa; no other collection number. ^4^ *tef1*: translation elongation factor 1-alpha; *tub2*: β-tubulin; *cmdA*: calmodulin; *his3*: histone H3. ^5^ N/A represents data that is not available.
