# Supplementary material for: Species Diversity, Mating Strategy and Pathogenicity of Calonectria Species from Diseased Leaves and Soils in the Eucalyptus Plantation in Southern China
Source: J Fungi (Basel). 2021 Jan 20;7(2):73. doi: 10.3390/jof7020073 (PMC7909555; doi:10.3390/jof7020073)
Supplement: Supplementary file 1 [file jof-07-00073-s001.pdf]

Supplementary Materials

Supplementary Figure S1. Phylogenetic tree of *Calonectria* species based on maximum likelihood (ML) analyses of the *tef1* gene sequences in this study. Bootstrap support values ≥ 70% are presented above the branches as follows: ML/MP. Bootstrap values < 70% and absent are marked with "*". Isolates highlighted in five different colours and bold were obtained in this study. Ex-type isolates are marked with "T". The "B" species codes are consistent with the recently published results in Liu and co-authors [1]. *Curvicladiella cignea* (CBS 109167 and CBS 109168) was used as an outgroup taxon.

Supplementary Figure S2. Phylogenetic tree of *Calonectria* species based on maximum likelihood (ML) analyses of the *tub2* gene sequences in this study. Bootstrap support values ≥ 70% are presented above the branches as follows: ML/MP. Bootstrap values < 70% and absent are marked with "*". Isolates highlighted in five different colours and bold were obtained in this study. Ex-type isolates are marked with "T". The "B" species codes are consistent with the recently published results in Liu and co-authors [1]. *Curvicladiella cignea* (CBS 109167 and CBS 109168) was used as an outgroup taxon.

Supplementary Figure S3. Phylogenetic tree of *Calonectria* species based on maximum likelihood (ML) analyses of the *cmdA* gene sequences in this study. Bootstrap support values ≥ 70% are presented above the branches as follows: ML/MP. Bootstrap values < 70% and absent are marked with "*". Isolates highlighted in five different colours and bold were obtained in this study. Ex-type isolates are marked with "T". The "B" species codes are consistent with the recently published results in Liu and co-authors [1]. *Curvicladiella cignea* (CBS 109167 and CBS 109168) was used as an outgroup taxon.

Supplementary Figure S4. Phylogenetic tree of *Calonectria* species based on maximum likelihood (ML) analyses of the *his3* gene sequences in this study. Bootstrap support values ≥ 70% are presented above the branches as follows: ML/MP. Bootstrap values < 70% and absent are marked with "*". Isolates highlighted in five different colours and bold were obtained in this study. Ex-type isolates are marked with "T". The "B" species codes are consistent with the recently published results in Liu and co-authors [1]. *Curvicladiella cignea* (CBS 109167 and CBS 109168) was used as an outgroup taxon.

Reference

1. Liu, Q.L.; Li, J.Q.; Wingfield, M.J.; Duong, T.A.; Wingfield, B.D.; Crous, P.W.; Chen, S.F. Reconsideration of species boundaries and proposed DNA barcodes for *Calonectria*. *Stud. Mycol.* **2020**, doi:10.1016/j.simyco.2020.08.001.

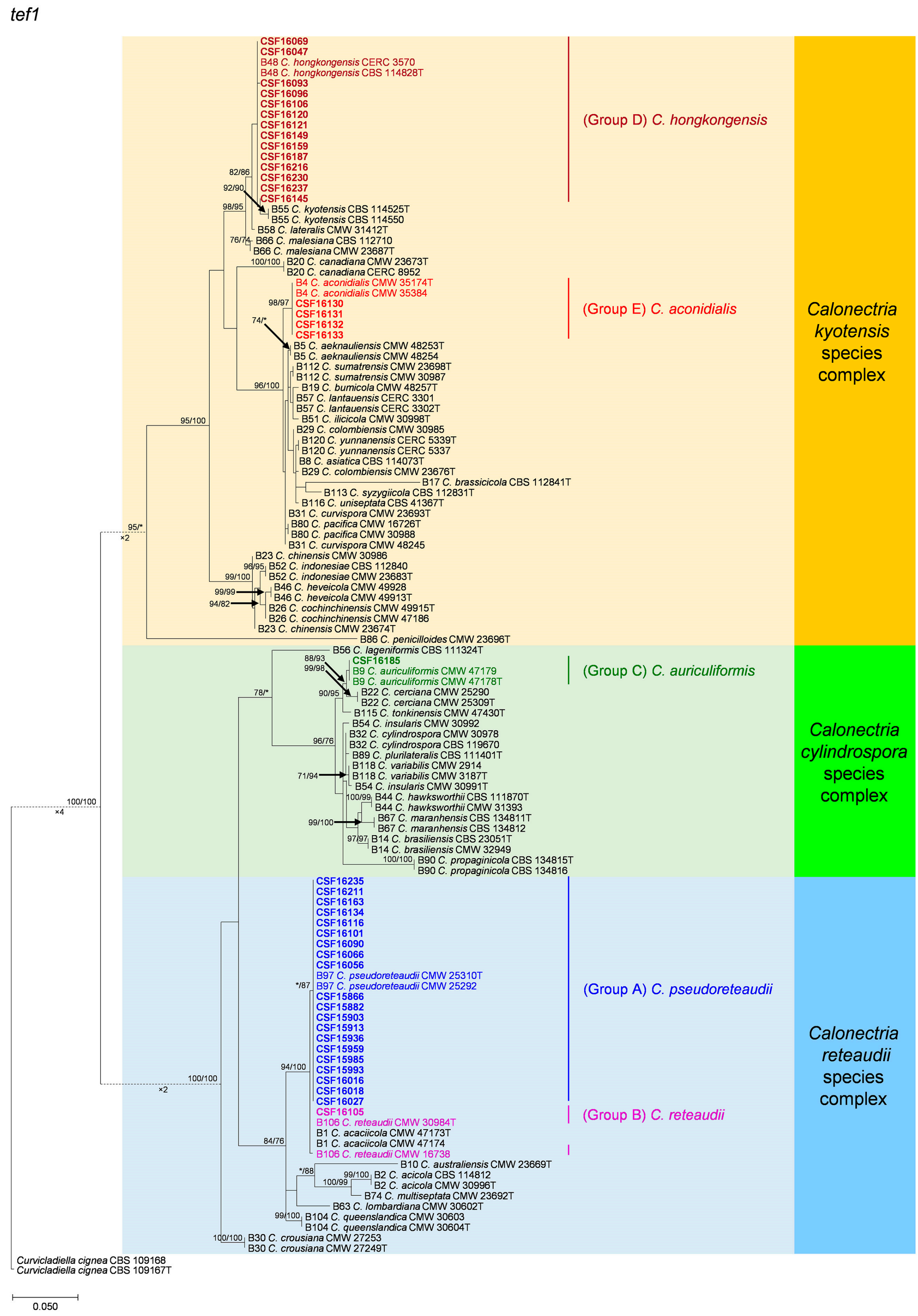

Supplementary Figure S1.

tub2

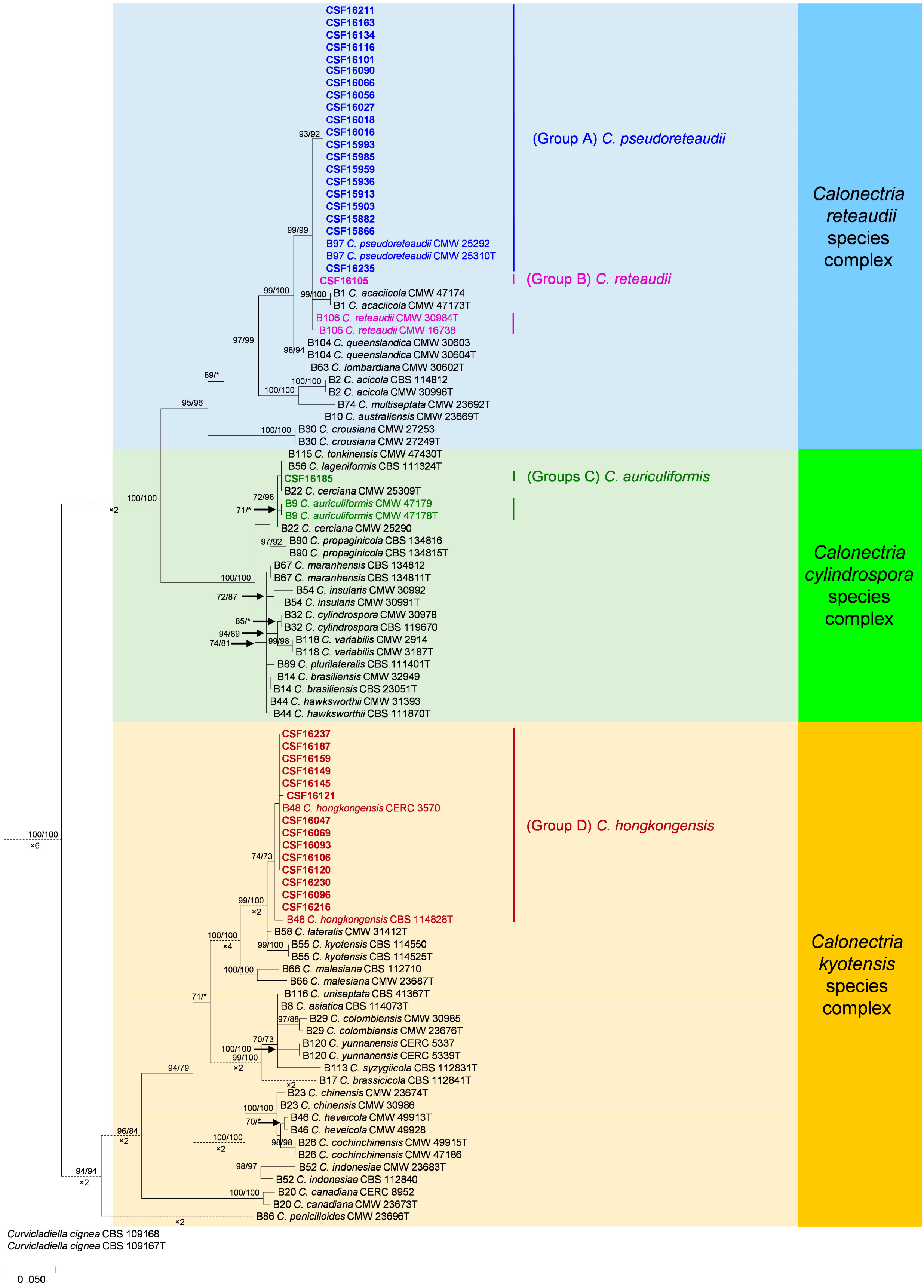

Supplementary Figure S2.

cmdA

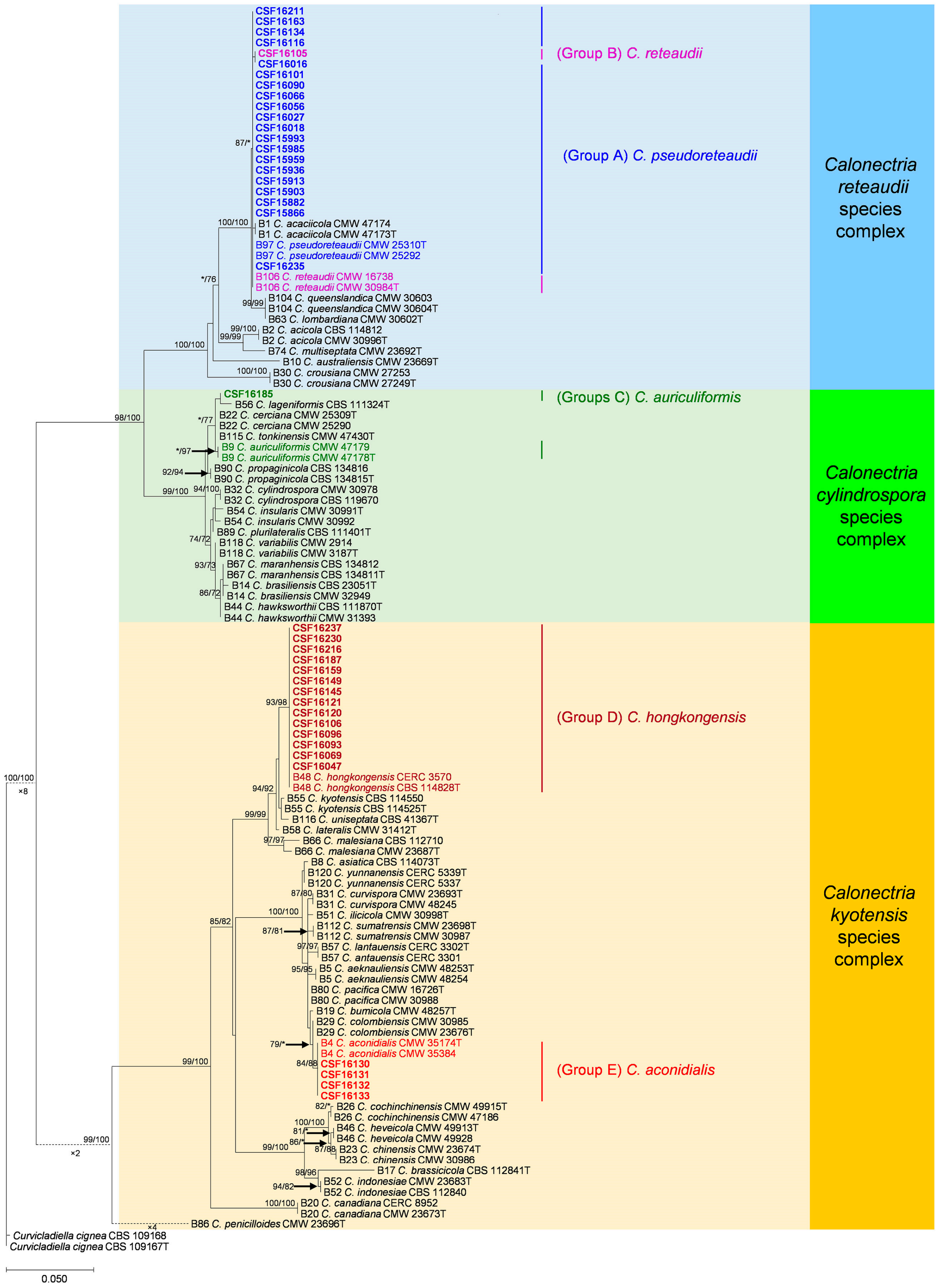

Supplementary Figure S3.

his3

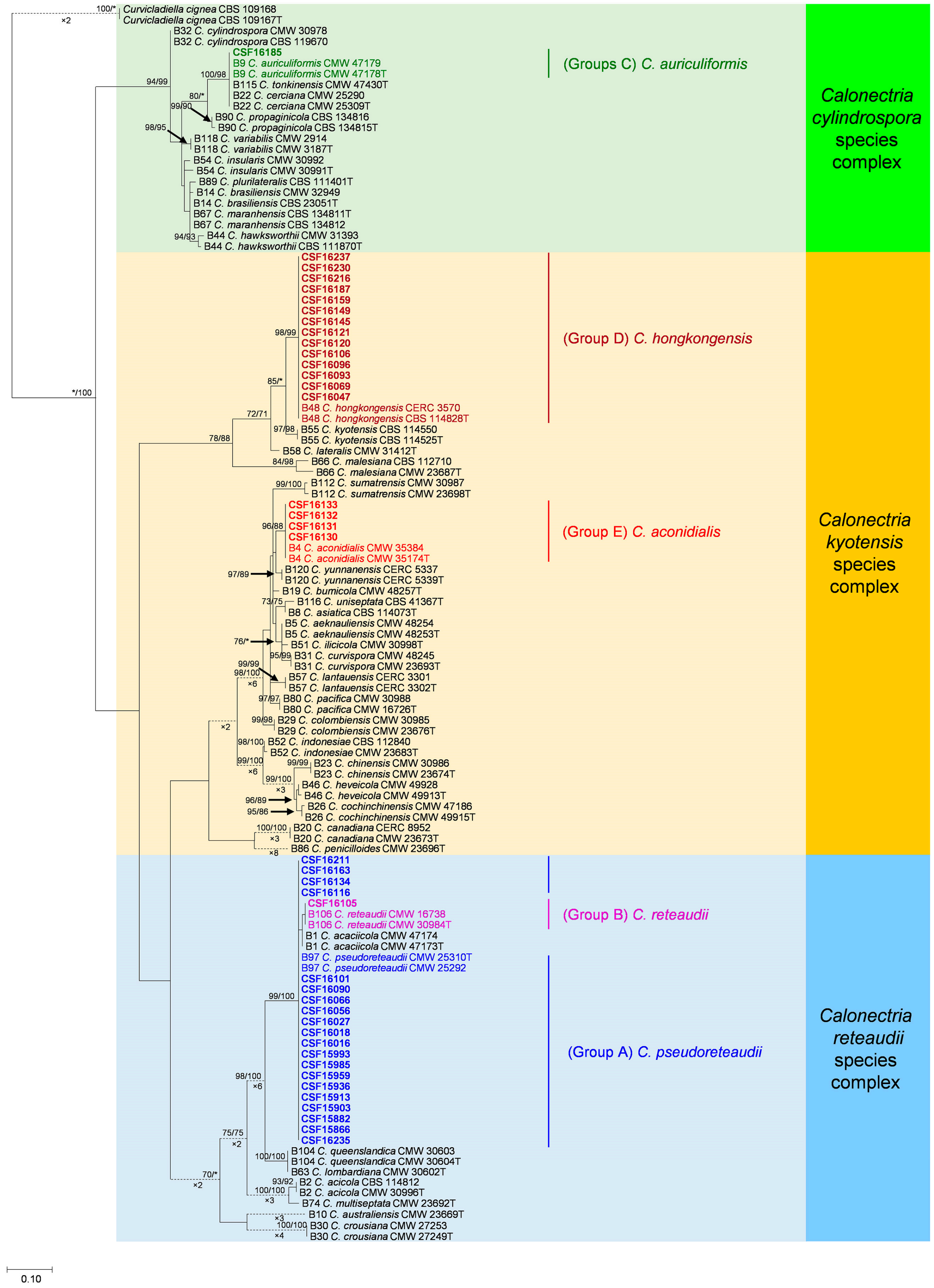

Supplementary Figure S4.
